# Epithelial tumor suppressor ELF3 is a lineage-specific amplified oncogene in lung adenocarcinoma

**DOI:** 10.1038/s41467-019-13295-y

**Published:** 2019-11-28

**Authors:** Katey S. S. Enfield, Erin A. Marshall, Christine Anderson, Kevin W. Ng, Sara Rahmati, Zhaolin Xu, Megan Fuller, Katy Milne, Daniel Lu, Rocky Shi, David A. Rowbotham, Daiana D. Becker-Santos, Fraser D. Johnson, John C. English, Calum E. MacAulay, Stephen Lam, William W. Lockwood, Raj Chari, Aly Karsan, Igor Jurisica, Wan L. Lam

**Affiliations:** 1 British Columbia Cancer Research Centre, Vancouver, BC Canada; 2 Department of Medical Biophysics, University of Toronto, Toronto, ON Canada; 3 Dalhousie University, Halifax, NS Canada; 4 Deeley Research Centre, Victoria, BC Canada; 5 Vancouver General Hospital, Vancouver, BC Canada; 6 Frederick National Lab for Cancer Research, Laboratory Animal Sciences Program, Frederick, MD USA; 7 Krembil Research Institute, University Health Network, Toronto, ON Canada

**Keywords:** Cancer, Cancer genetics, Cancer genomics, Cancer models, Lung cancer

## Abstract

Gene function in cancer is often cell type-specific. The epithelial cell-specific transcription factor ELF3 is a documented tumor suppressor in many epithelial tumors yet displays oncogenic properties in others. Here, we show that ELF3 is an oncogene in the adenocarcinoma subtype of lung cancer (LUAD), providing genetic, functional, and clinical evidence of subtype specificity. We discover a region of focal amplification at chromosome 1q32.1 encompassing the ELF3 locus in LUAD which is absent in the squamous subtype. Gene dosage and promoter hypomethylation affect the locus in up to 80% of LUAD analyzed. ELF3 expression was required for tumor growth and a pan-cancer expression network analysis supports its subtype and tissue specificity. We further show that ELF3 displays strong prognostic value in LUAD but not LUSC. We conclude that, contrary to many other tumors of epithelial origin, ELF3 is an oncogene and putative therapeutic target in LUAD.

## Abstract (web summary)

Tissue context can dictate why a gene can have seemingly opposing functions in different settings. ELF3 is tumor suppressive in many cancers of epithelial origin but in lung cancer, the authors describe an oncogenic role in the adenocarcinoma histology of non-small cell lung cancer.

## Introduction

There is increasing evidence of transcription factors exhibiting both tumor suppressive and oncogenic functions across cancers despite a common epithelial origin. For example, in lung cancer, the transcription factor Nuclear Factor I B (NFIB) has both tumor suppressive^1^ and oncogenic behaviors depending on the histological subtype^2,3^. Such dual functions are often attributed to DNA-level events specific to tumors arising from distinct cell types and lineages. Deleterious mutations in the epithelial-specific *E74 Like ETS Transcription Factor 3* (*ELF3*) have been described as tumourigenic in many epithelial tumors;^4–12^ in contrast, gene amplification and oncogenic activity has also been reported in others^13,14^. In lung cancer, the evidence suggests an oncogenic function as overexpression has been reported in non-small cell lung cancer (NSCLC) and a mouse lung tumourigenesis model^15–18^. Furthermore, the ELF3 locus is located within a region of recurrent DNA-level gain in non-small cell lung cancer on chromosome 1q32.1, suggesting a genetic mechanism of selection^19–21^. Contrary to many other recurrently gained or amplified regions in lung cancer, the gene target or targets of chromosome 1q gain remain elusive.

In this study, we have comprehensively analyzed 1835 human clinical samples of NSCLC and identify a disparate ELF3 expression pattern in the adenocarcinoma (LUAD) and squamous cell carcinoma (LUSC) histological subtypes. This overexpression is driven not only by a lineage-specific focal amplification event, but also via alternate DNA-level mechanisms, detected collectively in ~ 80% of LUAD and which hold prognostic value uniquely in LUAD. Functional experiments in cell and animal models confirm the oncogenic behavior of ELF3 in LUAD. We further discover ELF3 signaling networks to be highly tissue-specific, accounting for its conflicting functions across epithelial cancers. We demonstrate the duality of the ELF3 transcription factor and solidify its role as an oncogene in LUAD.

## Results

### ELF3 is frequently overexpressed in lung adenocarcinoma

**Fig. 1:**
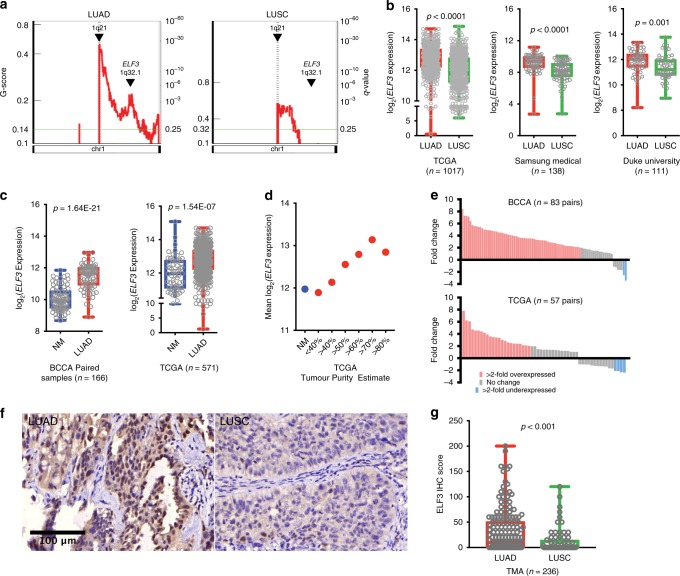
ELF3 is located within a region of focal amplification in lung adenocarcinoma. **a** Comparison of significantly focally amplified regions on chromosome 1q in LUAD and LUSC. **b** Comparison of *ELF3* expression between LUAD (red) and LUSC (green) in TCGA (*n* = 1017), Samsung Medical Centre (*n* = 138, GSE8894), and Duke University data sets (*n* = 111, GSE3141) by Mann–Whitney *U* test. **c** Box and whiskers plots of log_2_ *ELF3* expression in 83 paired cases of non-malignant lung (blue) and lung adenocarcinoma (red) in the BCCA cohort (Wilcoxon sign-rank test), and unpaired cases of 58 non-malignant lung and 513 lung adenocarcinoma samples in the TCGA cohort (Mann–Whitney *U* test). **d** Mean log_2_ *ELF3* expression in non-malignant lung (blue) and in LUAD (red) grouped by purity estimate in the TCGA cohort^55^. **e** Distribution of *ELF3* expression fold change in LUAD compared with paired non-malignant lung in the BCCA and TCGA cohort. **f** Representative ELF3 immunohistochemistry images from a tissue microarray of LUAD (left panel) and LUSC (right panel) (scale bar = 100 µm). **g** Box and whiskers plots of ELF3 immunohistochemistry (IHC) score as determined by pathologist review (*n* = 236, Mann–Whitney *U* test). Score was calculated by multiplying the percent of positive cells by the stain intensity (+1, +2, +3). In all box and whisker plots, center line represents the median, box bounds indicate the 25th and 75th percentiles, and whiskers extend from minimum to maximum. LUAD = lung adenocarcinoma, LUSC = lung squamous cell carcinoma, TMA = tissue microarray.

To investigate the oncogenic potential of ELF3 in NSCLC, we searched for recurrent DNA-level alterations that could indicate selection. *ELF3* mutations were uncommon in NSCLC, with a 1.4% mutation incidence in The Cancer Genome Atlas (TCGA) cohort (*n* = 408) (Supplementary Fig. 1a) and 1.0% in COSMIC v84 (*n* = 1302). We then searched for evidence of focal amplification at the *ELF3* locus and uncovered a narrow peak at 1q32.1 that encompassed *ELF3*, providing the first evidence of oncogenic selection. Interestingly, this focal amplification event was specific to LUAD histology, corroborating previous reports of lineage-specific frequent 1q gain in LUAD^19–22^ (Fig. 1a). This subtype specificity held true at the expression level, as *ELF3* expression was significantly higher in LUAD compared with LUSC in three independent cohorts (Fig. 1b, Supplementary Table 1). Immunohistochemistry data also showed elevated ELF3 protein expression in LUAD compared with LUSC (*n* = 236) (Fig. 1f, g, Supplementary Fig. 2). Similarly, *ELF3* expression was significantly positively correlated with LUAD lineage markers and negatively correlated with LUSC markers (Supplementary Table 2). When compared with adjacent non-malignant lung tissue, *ELF3* was significantly overexpressed in LUAD in the BC Cancer Agency (BCCA) (*n* = 83 pairs, Wilcoxon sign-rank *p* = 1.64E-21) and TCGA data sets (*n* = 571, Mann–Whitney *U* test *p* = 1.54E-07) (Fig. 1c), but not differentially expressed in LUSC (Supplementary Fig. 1b). Furthermore, TCGA LUAD samples with higher tumor cell purity exhibited higher *ELF3* expression, consistent with overexpression in tumor cells (Fig. 1d). In a pairwise analysis, greater than twofold overexpression was detected in 73 and 40% of BCCA and TCGA data, respectively (Fig. 1e).

### Genetic and epigenetic determinants of ELF3 overexpression

**Fig. 2:**
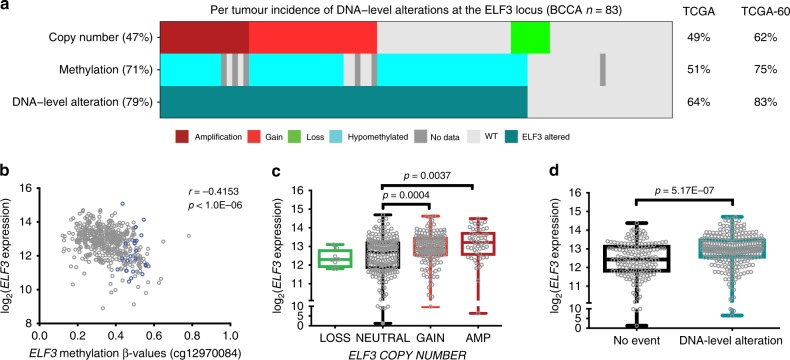
Genetic and epigenetic alterations at the *ELF3* locus. **a** Case-by-case representation of *ELF3* locus deregulation in the BCCA data set (*n* = 83 pairs) by genetic and epigenetic events. The frequencies of copy number gain or amplification, and promoter hypomethylation are indicated, as well as the cumulative frequency of *ELF3* DNA-level alterations in the BCCA data set. These frequencies are also summarized to the right of the plot for the TCGA (*n* = 420) and TCGA-60 (*n* = 252) data sets (TCGA cases with ≥ 60% tumor cellularity, see Methods). **b** Scatter plot of *ELF3* promoter methylation (*x* axis) and log_2_ *ELF3* expression (*y* axis) across 452 LUAD (gray) and 21 non-malignant lung (blue) samples (Spearman’s correlation). **c** Log_2_ *ELF3* expression as a function of DNA copy number alteration in LUAD (*n* = 420). *ELF3* expression is increased with activating events (Mann–Whitney *U* test). **d** Comparison of log_2_ *ELF3* expression between LUAD with gain and/or promoter hypomethylation (blue) compared with those without (black) (*n* = 420, Mann–Whitney *U* test). In all box and whisker plots, center line represents the median, box bounds indicate the 25th and 75th percentiles, and whiskers extend from minimum to maximum. LUAD = lung adenocarcinoma, AMP = amplification.

Focal *ELF3* amplification was detected in 14% of LUAD, and therefore could not explain the observed frequency of overexpression. Further investigations into broad copy number alterations and local promoter methylation changes that could explain additional cases of overexpression revealed disruption of the *ELF3* locus in ~ 80% of LUAD (BCCA = 79%, TCGA-60 = 83%) (Fig. 2a). *ELF3* expression correlated strongly with methylation of CpG probe cg12970084, as well as with gene dosage. Expression was significantly increased in tumors with genetic or epigenetic disruption compared to those without (Mann–Whitney *U* test *p* = 5.17E-07) (Fig. 2b–d, Supplementary Figs. 3, 4). A previous study identified SMAD4 as a direct transcriptional repressor of *ELF3*, and ERBB2 signaling as an activator of *ELF3* expression^17^. Given that ERBB2 is a known oncogenic driver of LUAD, we assessed the influence of inactivating *SMAD4* and activating *ERBB2* mutations on *ELF3* expression in tumors lacking locus disruption and find that *ELF3* expression was elevated in these samples (Mann–Whitney *U* test *p* = 0.06) (Supplementary Fig. 5). Therefore, ELF3 overexpression frequently observed in LUAD is explained predominantly by direct *ELF3* locus alterations and by mutations in upstream regulators.

### ELF3 disruption occurs across molecular subtypes

**Fig. 3:**
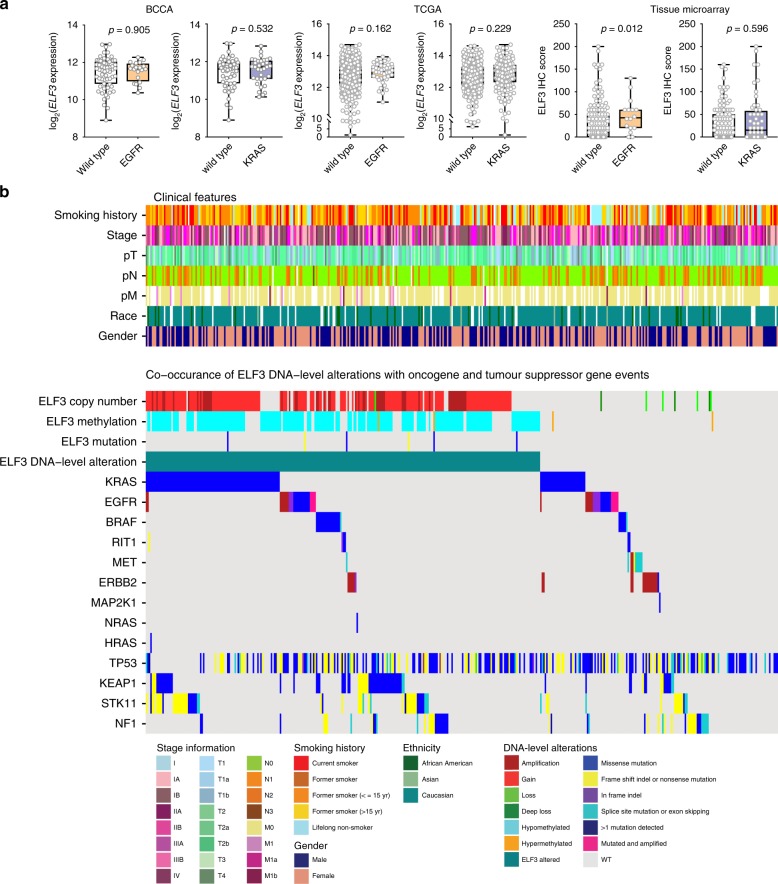
High ELF3 expression is not dependent on the molecular subtype of lung adenocarcinoma. **a** Comparison of log_2_ *ELF3* expression and ELF3 immunohistochemistry (IHC) score between cases with or without driver mutations in EGFR and KRAS (BCCA *n* = 83, TCGA *n* = 484, Tissue Microarray *n* = 161, Mann–Whitney *U* test). In all box and whisker plots, center line represents the median, box bounds indicate the 25th and 75th percentiles, and whiskers extend from minimum to maximum. **b** Case-by-case representation of clinical features (upper panel) and genomic alterations (lower panel) across 420 LUAD from the TCGA data set dichotomized into those with and without DNA-level alterations at the *ELF3* locus. Mutations and amplifications in prominent oncogenes and mutations in tumor suppressor genes are displayed.

As molecular cancer subtype information is valuable in guiding clinical management, we examined the effect of driver mutation status on ELF3 expression and searched for patterns of co-occurrence or mutual exclusivity at the DNA-level. High ELF3 expression occurred irrespective of KRAS or EGFR status in three independent cohorts (Fig. 3a). Similarly, we observed no association of ELF3 expression with KRAS, EGFR, or ALK in a panel of 25 LUAD cell lines (Supplementary Fig. 6). Mutations in known LUAD drivers^23^ (*KRAS, EGFR, BRAF, RIT1, ERBB2, MAP2K1, NRAS, HRAS, ERBB2 amplification, MET amplification*) and tumor suppressors (*TP53, KEAP1, STK11/LKB1, NF1*) occurred in both tumors with *ELF3* locus alterations and those without (Fig. 3b). There was a statistical enrichment of KRAS (Fisher’s exact *p* = 0.0011), STK11 (*p* = 0.0023), and KEAP1 (*p* = 0.0031) mutations in the *ELF3*-altered group, whereas *MET* and *ERBB2* mutations and amplifications were enriched in cases without *ELF3* disruption (*p* = 0.0009 and *p* = 0.0019, respectively). This is in agreement with previous associations of *KRAS* mutation and chromosome 1q gain, and correlations between *KRAS*, *STK11*, and *KEAP1* mutations^24,25^. Overall, we conclude that ELF3 holds biological relevance across LUAD molecular subtypes and proceed to pursue the oncogenic function of ELF3 function in representative cell models.

### ELF3 regulates cancer phenotypes of lung cell lines

**Fig. 4:**
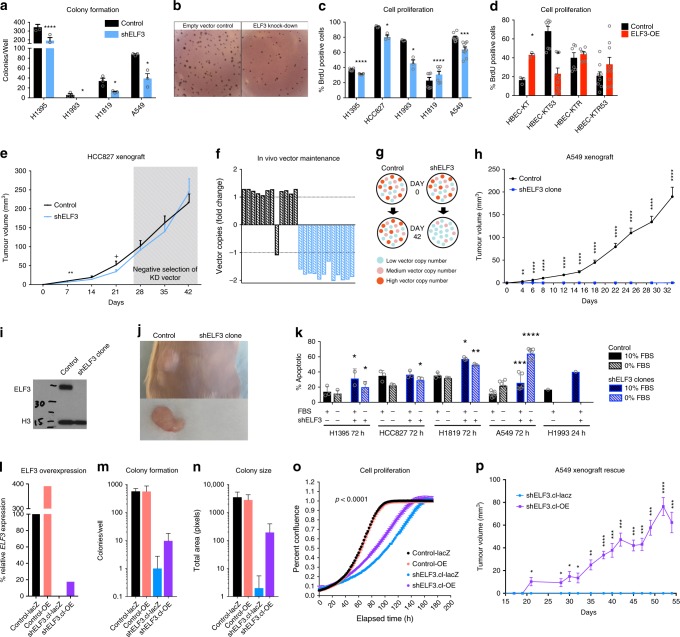
Manipulation of ELF3 expression regulates oncogenic phenotypes. Histogram summarizing the effect of shRNA-mediated ELF3 inhibition on **a**, **b** soft agar colony formation and **c** cell proliferation in lung adenocarcinoma cell lines, and **d** the effect of forced ELF3 overexpression (OE) on cell proliferation in HBEC-KT cell lines with and without p53 knockdown (HBEC-KT53) and/or induction of KRAS (HBEC-KTR, HBEC-KTR53). **e** Tumor growth of isogenic HCC827 shELF3 and control cells in NOD-SCID mice (*n* = 12). **f** Quantification of shELF3 and control vector DNA copy number in endpoint tumors compared with input material. **g** Putative model of clonal drift throughout xenograft tumor growth. At Day 0 we assume a mixed population of cells with low (blue), medium (pink), and high (orange) vector copy numbers (CN) for both populations. Over time, the relative proportion of low shELF3 vector CN clones dominates owing to the growth advantage provided by ELF3 expression. **h** Tumor growth curve of clonal populations of isogenic A549 shELF3 and control cells in NOD-SCID mice (*n* = 24). **i** ELF3 expression of input clones as measured by immunoblot. **j** Control and absent shELF3 tumors at endpoint. **k** Cell viability as measured by annexin/PI staining of control (black bar) and clonal shELF3 (blue bar) LUAD cell lines cultured in complete (10% FBS, solid fill) or serum starved (0% FBS, hatched fill) media (H1993 *n* = 1 replicates owing to viability issues). **l** qPCR of *ELF3* mRNA expression in control and shELF3 clonal cell lines with forced ELF3-OE or empty vector control (lacZ). Histograms summarizing the effect of ELF3 overexpression on colony formation, in terms of number **m** and size **n** of colonies formed (paired two-tailed Student’s *t* test). **o** Cell proliferation shows rescued growth rate with ELF3-OE in A549 cells (Wilcoxon test). **p** Tumor growth of shELF3.cl-lacZ and shELF3.cl-OE A549 cells in NRG mice (*n* = 8). In all box and whisker plots, center line represents the median, box bounds indicate the 25th and 75th percentiles, and whiskers extend from minimum to maximum, all histograms display the mean + SEM (*n* = 3 biological replicates unless otherwise stated). CN = copy number, +*p* < 0.10, *p < 0.05, **p < 0.01, ***p < 0.001, ****p < 0.0001, paired two-tailed Student’s *t* test.

ELF3 expression was stably inhibited by lentiviral-mediated delivery of five shRNA vectors in the LUAD cell line, HCC827, which exhibits high ELF3 expression. The optimal two shRNAs were identified, and cell viability was assessed by MTT assay. ELF3 knockdown (shELF3) cell lines demonstrated significantly reduced viability as compared with their isogenic empty vector controls (Supplementary Fig. 7a, b). With this preliminary observation that ELF3 knockdown reduces oncogenic phenotypes, we expanded our experiments to include four additional cell lines harboring diverse molecular drivers (A549, H1395, H1993, and H1819 (Supplementary Fig. 7c, d)). For subsequent molecular assays, the shRNA with the highest degree of knockdown was selected and compared with empty vector control (HCC827 shRNA-1, A549 shRNA-1, H1395 shRNA-5, H1993 shRNA-5, H1819 shRNA-5). Four of five lines with ELF3 knockdown (shELF3) exhibited reduced proliferation as measured by BrdU incorporation assay, and all evaluable lines demonstrated a significantly reduced ability to form colonies in soft agar (Fig. 4a–c). To account for potential off-target effects, we validated the effect of ELF3 knockdown on cell viability using an siRNA pool (five siRNAs) on A549 cells and further tested specificity by including a cell line with low ELF3 expression (H2030). This observation was specific to LUAD cell lines with high ELF3 expression, as viability of A549 cells was significantly decreased while H2030 cells were not affected by ELF3 siRNA-mediated inhibition (Supplementary Fig. 7f, g).

To examine the impact of ELF3 overexpression in a non-malignant setting, ELF3 was stably overexpressed (ELF3-OE) in immortalized but untransformed human bronchial epithelial cells (HBEC-KT), which do not normally express ELF3, and compared to cells transformed with an empty vector control (Supplementary Fig. 7e). Consistent with shELF3, ELF3-OE cells displayed significantly increased proliferation (Fig. 4d), but overexpression alone was insufficient to transform cells in soft agar colony formation assays. As cell transformation requires multiple oncogenic manipulations, we assessed the ability of ELF3 to transform HBEC-KTs in the background of additional molecular alterations. We stably overexpressed ELF3 in HBEC-KTs with p53 knockdown (HBEC-KT53), with oncogenic RAS expression (HBEC-KTR), and with both alterations (HBEC-KTR53), again comparing to empty vector control^26^. We first assessed proliferation as above and found ELF3 overexpression to significantly decrease the proliferative capacity of HBEC-KT53 cells, whereas no significant difference was observed in HBEC-KTR or HBEC-KTR53 cells (Fig. 4d). We then assessed cell transformation by colony formation assay. No sizable colonies were detected in any condition following three biological replicates, leading us to conclude ELF3 is unable to transform HBEC-KT cells with these specific molecular alterations in vitro. It is evident that ELF3 is capable of regulating oncogenic phenotypes; however, these results indicate the functional importance of ELF3 may arise during a later stage of tumor development or on a background of further genetic alterations.

### Clonal elimination of ELF3 abolishes tumor growth

The effect of ELF3 inhibition on tumor growth was examined in vivo using a xenograft model of HCC827 shELF3 (shRNA-1) cells and isogenic controls (*n* = 12 NOD-SCID mice). Although growth of polyclonal shELF3 tumors was initially slowed, it eventually reached control levels (Fig. 4e). Analysis of endpoint tumors showed that knockdown was not maintained (Supplementary Fig. 8a), and clones within the shELF3 tumors that had fewer copies of the shELF3 vector (and higher ELF3 expression) out-competed clones with increased copies of shELF3 vector (Fig. 4f–g). This suggests strong selective pressure maintains ELF3 expression throughout xenograft tumor growth.

Based on the tendency of polyclonal shELF3 cell populations to restore ELF3 expression, we established clonal shELF3 and controls for all cell lines and selected the best knockdown clones for molecular assays. Interestingly, the shELF3 clones were morphologically distinct and demonstrated reduced viability from their control counterparts (Fig. 4k, Supplementary Fig. 9). The effect of ELF3 inhibition on tumor growth was once again assessed using clonal populations of cells (A549 shRNA-1, and isogenic control) injected into the flanks of NOD-SCID mice (*n* = 24). Clonal A549 shELF3 cells did not express detectable levels of ELF3 at time of injection (Fig. 4i, Supplementary Fig. 8b). Control xenografts formed a large tumor mass over time, whereas shELF3 clones remarkably showed no evidence of growth over the course of the experiment (Fig. 4h–j). At endpoint, small shELF3 masses were identified in six mice (25%). These small masses had restored human *ELF3* expression to control levels, indicating the strong selective pressure and requirement for ELF3 in this xenograft model. This role in tumor initiation is functionally reminiscent of a previously established role in regulating stemness via NOTCH3^27^. However, we did not detect NOTCH3 upregulation, implicating alternative signaling networks (Supplementary Fig. 8b–e). Nevertheless, we establish the requirement of ELF3 expression for tumor initiation and growth.

The potential for off-target effects was investigated by rescuing ELF3 expression in our clonal A549 shELF3 cell lines; in addition, ELF3 was overexpressed in the corresponding clonal control. This overexpression resulted in a 20% rescue in the shELF3 cells, which restored the morphology to the control state, and a fourfold increase relative to control cells (Fig. 4l, Supplementary Fig. 10). The effect of ELF3 overexpression in control A549 cells expressing ELF3 was modest and did not significantly affect colony formation but increased cell proliferation (Wilcoxon *p* < 0.0001). In contrast, ELF3 rescue in A549 shELF3 clones increased the size and number of colonies formed, and the proliferation rate of cells (Fig. 4m–o). The influence of ELF3 overexpression on A549 control and clonal shELF3 xenograft growth was assessed by subcutaneous flank injection (*n* = 8 mice). Although ELF3 overexpression in A549 control cells did not significantly increase xenograft tumor growth, the modest ELF3 rescue in clonal A549 shELF3 cells restored the ability to establish tumors in vivo (Fig. 4p, Supplementary Fig. 10). Interestingly, ELF3 expression in tumors at endpoint had increased eightfold compared with the injected cell lines, providing further evidence of its importance to in vivo growth (Supplementary Fig. 10). These results confirm the requirement of ELF3 for A549 xenograft growth and further support its role as an oncogene in LUAD.

### ELF3 is associated with broad transcriptional reprogramming

**Fig. 5:**
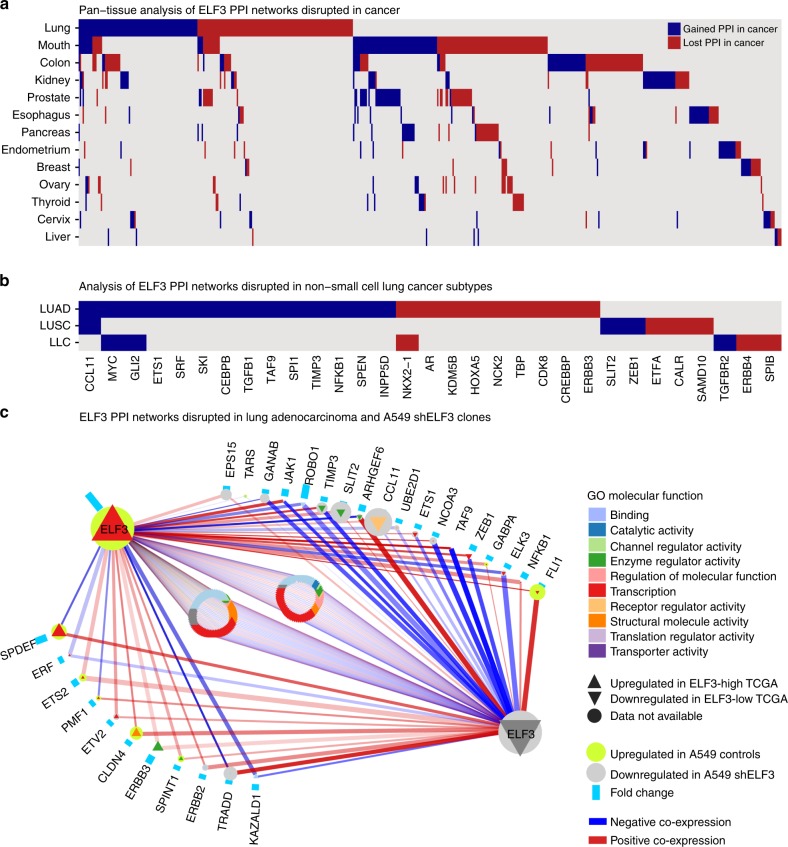
Pan-cancer analysis of ELF3 protein–protein interaction networks reveals tissue specificity. **a** Tissues are ordered vertically according to decreasing number of altered ELF3 PPIs. Disrupted PPIs were identified by comparing cancer profiles with non-malignant profiles in the same tissue types, with PPIs gained in cancer shown in red, and those lost in cancer shown in blue. The largest disrupted network was in lung cancer, with 111 PPIs gained and 85 PPIs lost in cancer. Considering all altered PPIs, 57% were specific to one tissue and only 15 PPIs were gained or lost in more than three tissues. **b** The largest disrupted network was in lung adenocarcinoma (LUAD), with 14 lost and 9 gained PPIs in cancer. The only altered PPI common to LUAD and squamous cell carcinoma (LUSC) was CCL11, whereas MYC, GLI2, and NKX2–1 were common to LUAD and large cell carcinoma (LULC). The protein names of the altered PPIs are indicated at the bottom of the figure. **c** Comparison of altered ELF3 PPIs between TCGA LUAD data and isogenic A549 cells (KRAS mutant samples only). Twenty-nine out of 156 PPI partners were significantly deregulated in LUAD with high ELF3 expression and are color-coded by their respective GO Molecular Function. Proteins at the left-side show significantly upregulated (up-triangles) mRNAs when ELF3 is highly expressed in TCGA, and right-side proteins represent significantly downregulated (down-triangles) mRNAs when ELF3 is highly expressed in TCGA. Edge color represents positive co-expression (red edges) or negative co-expression (blue edges), and edge thickness is proportional to the number of data sets supporting the edge. Node outline color represents up- or down-regulation in A549 control cells compared with clonal shELF3 cells (*n* = 3 biological replicates). Therefore, bright green outline around up-triangles, and gray outline around down-triangles indicate consistency between TCGA and isogenic cell line data. Node size is proportional to the total number of altered PPIs, and node highlight size is proportional to the FDR corrected *p* value of expression fold change in isogenic cell lines, whereas blue bars indicate the fold change value. Circles in the center indicate ELF3 partners whose differential expression was not statistically significant. PPI = protein–protein interaction.

Our results point to a clear oncogenic role in LUAD, countering classifications of ELF3 as a tumor suppressor gene in other epithelial tissues^12^. With mixed reports of gene behavior, we sought to define the tissue specificity of ELF3 interaction networks. We leveraged gene expression profiles of 13 tissues to construct ELF3 protein–protein interaction (PPI) networks, and assessed their disruption in a pan-cancer analysis^28^. We discovered remarkable tissue specificity of ELF3 PPI networks, with the largest disrupted network observed in lung (Fig. 5a, Supplementary Fig. 11). A NSCLC-specific analysis indicated the majority (25/33) of ELF3 PPI disruptions occurred in LUAD as compared with LUSC and lung large cell carcinoma (Fig. 5b) prompting a detailed investigation of altered PPIs and pathways in this subtype^29^.

ELF3 PPI networks disrupted by high ELF3 expression were analyzed in LUAD as compared with non-malignant lung tissue, and LUAD dichotomized by KRAS mutation status based on the DNA-level association of ELF3 disruption in KRAS mutant LUAD. A total of 69 deregulated PPIs were supported by at least one analysis (Supplementary Figs. 12–14, Supplementary Data 2). PPIs that were supported across all analyses included ERBB3, ETS1, and TIMP3, whereas those supported by at least two analyses included ARHGEF6, CCL11, CLDN4, ELK3, ERBB2, FLI1, GLI2, KDM5B, NFKB1, PMF1, ROBO1, SLIT2, SPDEF, SPI1, SPINT1, TAF9, TRADD, ZEB1, androgen receptor (AR), and INPP5D. Importantly, ELF3 PPI network data from KRAS mutant LUAD was compared against whole-genome expression data generated from isogenic A549 control and shELF3 clonal cell lines. Of the proteins with deregulated PPIs identified in TCGA, 26 were available on the A549 gene expression microarray. Twenty-one out of these 26 demonstrated significant levels of differential expression as a result of isogenic ELF3 expression manipulation and were considered validated (Fig. 5c, Supplementary Data 2).

Pathway analysis identified deregulation of not only those consistent with previously established ELF3-related signaling events—for example, IL-1β, NFκB, p38, and JNK signaling in inflammation^30–33^, ETS transcription factors in MAPK signaling^34,35^, and NOTCH and WNT in cancer stem cells and colorectal cancer^14,27,36^—but other pathways that agreed with phenotypes established in our isogenic systems and pointed to previously undescribed functions. These included cell cycle, apoptosis, adhesion, and motility functions (organization, junction, adherens, cadherin), as well as AR signaling (Supplementary Fig. 12 and Supplementary Data 1). Interestingly, AR-ELF3 was a LUAD-specific interaction, and high expression of AR has been reported in NSCLC and associated with growth potential in murine models^37^ (Supplementary Fig. 13).

Moreover, our network analyses predict other transcription factors as predominantly altered ELF3-binding partners in LUAD (45–56%), including the LUAD lineage-specific oncogene NKX2–1 (PPI in LUAD)^38^ and epithelial-to-mesenchymal transition-promoting ZEB1 (anti-correlated with ELF3 expression) (Fig. 5, Supplementary Figs. 13, 14 and Supplementary Data 2). Similarly, we find positive associations between ELF3 and E-Cadherin, an epithelial marker, in our isogenic cell models and TCGA protein expression data (Supplementary Fig. 15). ELF3-altered partners have varied subcellular localization, raising the question of ELF3 function in alternative cellular compartments. We investigated ELF3 localization in human tumors (immunohistochemistry) and cell lines (immunofluorescence) and observe both nuclear and cytoplasmic localization (Supplementary Figs. 2, 16). It is possible ELF3 has as-of-yet uncharacterized functions, such as its ability to transform breast cells through an unknown cytoplasmic mechanism^39^.

### High ELF3 expression is associated with poor survival

**Fig. 6:**
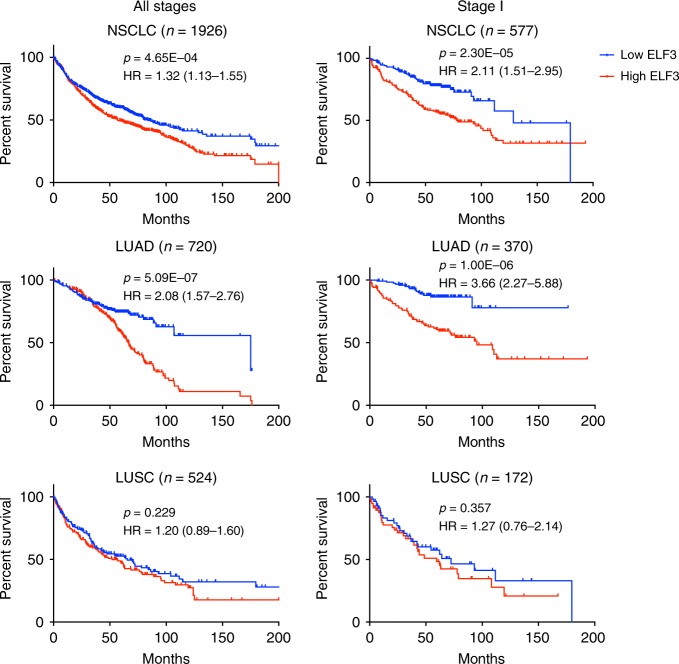
Prognostic relevance of ELF3 expression in non-small cell lung cancer. Kaplan–Meier survival curves comparing overall survival of lung cancer patients with high or low ELF3 expression (top and bottom tertiles, log-rank *p* values). All Stages: NSCLC *n* = 1926, *p* = 4.65E-04, Hazard Ratio (HR) (95% confidence interval) = 1.32 (1.13–1.55); LUAD *n* = 720, *p* = 5.09E-07, HR = 2.08 (1.57–2.76); LUSC *n* = 524, *p* = 0.229, HR = 1.20 (0.89–1.60). Stage I: NSCLC *n* = 577, *p* = 2.30E-05, HR = 2.11 (1.51–2.95); LUAD *n* = 370, *p* = 1.00E-06, HR = 3.66 (2.27–5.88); LUSC *n* = 172, *p* = 0.357, HR = 1.27 (0.76–2.14). NSCLC = non-small cell lung cancer; LUAD = lung adenocarcinoma; LUSC = lung squamous cell carcinoma.

Finally, we investigated the prognostic utility of ELF3 in NSCLC (*n* = 1715)^40^. High *ELF3* expression was associated with poor overall survival (OS) in NSCLC patients (log-rank *p* = 4.65E-04), an association which improved in Stage I patients (log-rank *p* = 2.30E-05) (Fig. 6). However, our study supports an increased biological relevance in the LUAD subtype of NSCLC. Agreeing with this, high *ELF3* expression was highly significantly correlated with OS in LUAD (log-rank *p* = 5.09E-07; Stage I *p* = 1.00E-06), whereas no significant distinction was observed in LUSC (Fig. 6). These data further demonstrate the clinical relevance of ELF3 expression in LUAD.

## Discussion

Here, we find that ELF3 is overexpressed in LUAD compared with non-malignant lung and LUSC, and promotes oncogenic phenotypes including the requirement for tumor growth in vivo. These data refine  a recent study implicating ELF3 as an oncogene in NSCLC^18^, uncovering DNA-level mechanisms driving overexpression and a strong survival association specifically in the LUAD histology. Interestingly, the low mutation rate of *ELF3* in lung cancer has allowed it to elude sequencing-based screens of recurrently altered oncogenes; the high frequency of alternative DNA-level disruptions in upwards of 80% of LUAD underscores the importance of interrogating multiple ‘omics' levels. Furthermore, this ETS transcription factor appears to follow divergent paths in cancer with contrasting genetic mechanisms of disruption. Although behavior and alteration mechanisms similar to LUAD, including gene amplification, have been observed in colorectal and breast cancer^13,14,39^, contrasting recurrent deleterious mutations in ELF3 have been identified in biliary tract cancer, mucinous ovarian carcinoma, and cancers of the cervix, stomach, and bladder, indicating a tumor suppressive role in these cancer types^4–8^.

This observed diversity across epithelial malignancies is further exacerbated by highly tissue-specific co-expression networks revealed in our pan-cancer analysis. Interestingly, the largest ELF3-related network was identified in lung cancer, more specifically LUAD. This could point to not only the significant functional role in malignant LUAD but also a larger biological role in normal lung biology. Although ELF3 is currently poorly characterized in this regard, what is known ties ELF3 to both fetal lung development and airway tissue repair, functions that are often co-opted by cancer cells. *ELF3* is highly expressed in human fetal lung tissue^41^, and in mice *Elf3* knockout induces embryonic lethality^42^. In the adult airway, Elf3 regulates the kinetics of tissue repair following Clara cell-specific injury, which is a putative cell-of-origin for LUAD^43^. Mirroring these studies, we find that ELF3 cancer-specific networks include NXK2–1, a regulator of fetal lung development and oncogenic potential in LUAD^38^. We also find ELF3 regulates proliferation and apoptosis both experimentally and through pathway analysis. This broad reprogramming of ELF3-centric expression networks in LUAD underpins its significance to this disease subtype and is representative of large-scale reprogramming of cell states that result from the deregulation of transcription factors^44–46^. Indeed, the development of therapeutic strategies to inhibit oncogenic transcription factors such as ETS family members is an area of active research^47^. The specific relevance of ELF3 to LUAD is highlighted by its subtype-specific association with poor patient outcome; however, we caution that ELF3 alterations are enriched in KRAS, STK11, and KEAP1 mutant tumors, all of which are themselves associated with poor prognosis. Further studies are warranted to uncouple the effects of these genetic alterations and to define the mechanistic role of ELF3 in LUAD which may clarify its association with patient survival.

Chromosome 1q is a region of recurrent gain in NSCLC that is most prominently associated with LUAD and disease aggressiveness^19–22^. Although putative oncogenic targets of this event have been proposed, no conclusive oncogene in this region has been identified. These putative gene targets have been found to regulate complementary tumor hallmarks including autophagy and immune phenotypes^48–50^. Our discovery of a region of focal amplification at the ELF3 locus, coupled with our findings that ELF3 knockdown is highly selected against in a polyclonal tumor growth model and completely abrogates tumor growth in a clonal setting highlights the importance of this oncogene. Importantly, we further discover additional mechanisms of ELF3 overexpression including DNA copy number gain, promoter hypomethlyation, and mutations in upstream regulators in up to 80% of LUAD and across molecular subtypes. Based on the strong phenotype observed in our in vivo models, the potential for broad applicability to the most common subtype of LUAD, and the association of improved survival for patients with low *ELF3* expression, the feasibility of therapeutic inhibition should be explored.

## Methods

### Patient tissue accrual

Tumor and non-malignant lung tissues from the British Columbia Cancer Agency (BCCA) cohort were collected from treatment-naive patients at the time of surgical resection and frozen in liquid nitrogen. Tissues were obtained from the Tumor Tissue Repository of the British Columbia Cancer Agency or Vancouver General Hospital under informed written patient consent, relevant ethical regulations, and with approval from the University of British Columbia—BC Cancer Agency Research Ethics Board. Hematoxylin and eosin staining was performed for each malignant and non-malignant sample and reviewed by a pathologist. Subsequently, tumor specimens were microdissected to contain at least 70% tumor cell content and non-malignant samples were verified to be histologically normal. DNA and RNA from alternating sections were extracted using standard protocols. Tissue microarrays from the Dalhousie University cohort were made from surgically resected lung cancer specimens from treatment-naive patients under informed written patient consent and with approval from the Nova Scotia Health Authority Research Ethics Board.

### Immunohistochemistry

Formalin-fixed paraffin embedded (FFPE) tissues were deparaffinised, and antigen retrieval was performed in decloaking chamber plus with Diva decloaker (Biocare Medical, Pacheco, CA, USA). Endogenous peroxidise blocking with peroxidazed-1 (Biocare Medical) and non-specific blocking with background sniper 1 (Biocare Medical) was performed in the Intellipath FLX. Next, primary antibody (1:75 dilution, anti-ELF3 HPA003479, Sigma-Aldrich, Oakville, ON, Canada) was added, followed by Rb-HRP polymer and DAB chromogen (Biocare Medical). Slides were counterstained with CAT hematoxylin (1:5 dilution, Biocare Medical). Slides were imaged on a Pannoramic Digital Slide Scanner (3D Histotech) and image analysis conducted using Pannoramic Viewer.

### Tissue profiling

Gene expression profiles of 83 paired LUAD and NM tissues were generated on the Illumina HT-12 Whole Genome 6, v3 BeadChip array according to the manufacturer’s instructions (Illumina, San Diego, CA, USA). Bead-level data were pre-processed using the R package mbcb (ver. 2.11.0) to perform background correction and probe summarization. Data were then quantile normalized and log_2_ transformed. Pairwise analysis of log_2_expression values comparing LUAD and NM was performed using the Wilcoxon sign-rank test. Fold change was calculated by subtracting the normal log_2_(expression) value from the paired malignant log_2_(expression) value, and transforming the log_2_(fold change) value: Fold change = 2^(log_2_(fold change)). Paired LUSC and NM tissues were obtained as described for LUAD. Extracted RNA was subjected to RNA sequencing on the Illumina HiSeq 2000 sequencing platform following library construction and bar-coding using a plate based protocol developed at the British Columbia Genome Science Centre^51^. For DNA copy number analysis, DNA was hybridized to Affymetrix Genome-Wide Human SNP Array 6.0 arrays according to the manufacturer’s instructions (Affymetrix, Santa Clara, CA, USA). Raw CEL probe intensity files were processed and normalized using Partek Genomics Suite 6.5. Probe sequence, fragment length, GC content, and background adjustments were applied to correct for biases in signal intensities. Tumor copy number profiles were processed using the corresponding non-malignant copy number profile as a baseline. Thresholds for DNA copy number alterations were applied as follows: copy number loss < 1.7, copy number gain > 2.3. For methylation analysis, DNA was bisulfite converted and hybridized to the Illumina Infinium Human Methylation 27 array. Raw methylation data were corrected for color bias and normalized using SSN normalization with the Bioconductor package lumi in R statistical computing software. Probe hypermethylation was defined as a change in *β*-Value (∆βV) ≥ 0.15 (at least 15% more methylated in tumor), whereas probe hypomethylation was defined as a ∆βV ≤ −0.15 (at least 15% less methylated in tumor). A two-group comparison between LUAD and NM was performed for each probe of interest using a Mann–Whitney *U* test. For all statistical analyses, a *p* value of < 0.05 was considered significant.

### The Cancer Genomic Atlas data

For DNA copy number analysis, level 3 whole- genome SNP 6.0 copy number segmentation files (GRCg37/hg19) were downloaded from the TCGA Data Portal https://tcga-data.nci.nih.gov/tcga/tcgaDownload.jsp. Specifically, 551 LUAD (tissue codes 01 and 02) *.nocnv_hg19.seg.txt files, from which a fixed set of germline-variable probes have been removed, were assembled into a master segmentation file for GISTIC 2.0 analysis^52^. GISTIC 2.0 analysis was run on the Broad server using the following Hg19 URL marker file: ftp://ftp.broadinstitute.org/pub/GISTIC2.0/hg19_support/genome.info.6.0_hg19.na31_minus_frequent_nan_probes_sorted_2.1.txt. Amplification and deletion thresholds were increased to 0.3; default settings were used for all other parameters. For gene expression analysis, level 3 LUAD gene expression (IlluminaHiSeq) data from 513 LUAD and 58 NM lung tissue specimens was downloaded from CancerBrowser (https://genome-cancer.ucsc.edu/proj/site/hgHeatmap/). Fifty-seven LUAD cases had paired NM expression. Fold change was calculated with a cutoff of ±2, and expression was compared using a paired sign-rank test. For methylation analysis, level 3 Illumina Infinium HumanMethylation450K data from 460 LUAD and 32 NM specimens was downloaded from CancerBrowser. For gene mutation analysis, level 2 Mutation Annotation Format (MAF) files for 543 LUAD were downloaded from the TCGA Data Portal. For the genes *KRAS*, *BRAF*, *EGFR*, *ERBB2*, *MAP2K1*, *MET*, *HRAS,* and *NRAS*, only annotated driver mutations were considered^23^. For all other genes analyzed, silent mutations were removed. For protein expression analysis, TCGA normalized protein expression data for E-cadherin was downloaded from cBioPortal^53,54^. Lung tumor specimens from The Cancer Genome Atlas are composed of a varying degrees of tumor cell contents^55^. Owing to potential dilution of expression or methylation signals by stromal cells, a second TCGA data set was generated that comprised of samples with at least 60% tumor cell content, termed the TCGA-60 data set for tumor purity analysis.

### Cell line experiments

Cell lines were obtained from American Type Culture Collection (ATCC, Manassas, VA, USA) and maintained according to ATCC guidelines. All cell lines were routinely monitored for mycoplasma contamination. LUAD cell lines were cultured in RPMI 1640 (Gibco–ThermoFisher, Waltham, MA, USA) supplemented with 10% FBS and maintained in a humidified 5% CO_2_ incubator. Human Bronchial Epithelial Cells are untransformed cells that have been immortalized by expression of cyclin-dependent kinase (Cdk) 4 and humantelomerase reverse transcriptase (hTERT) (HBEC-KT), and have been further altered to harbor p53 knockdown, oncogenic RAS expression (HBEC-KTR), and with both alterations (HBEC-KTR53)^26,56^. All HBEC-KT cells were cultured in Opti-MEM media (Gibco–ThermoFisher, Waltham, MA, USA) supplemented with 0.0002  ng/μl EGF and 30 μg/ml BPE. Stable knockdown of ELF3 was achieved by lentiviral transfection of vectors encoding shRNA inserts directed against ELF3 mRNA as well as a puromycin resistance selectable marker (Sigma-Aldrich). Five shRNA clones were tested and the clone with the best knockdown of ELF3 transcript and protein levels in each cell line was selected for phenotypic assays. Virus was prepared for ELF3-shRNAs and a control with no shRNA insert. LUAD cell lines were transfected, and after 24 h media was replaced with puromycin-containing media. Following complete puromycin-induced death of control cells after 3–5 days, transfected cells were cultured in puromycin-media for an additional 7 days. Knockdown efficiencies were quantified by qRT-PCR (TaqMan—Applied Biosystems, Carlsbad, CA) using 18 S as an endogenous control: Hs00963881_m1 (ELF3) and Hs99999901_s1 (18 S), and verified at the protein level by western blot (described below). Stable overexpression of ELF3 in HBEC-KT and A549 cell lines was achieved by lentiviral delivery of a vector containing the full ORF with a blasticidin resistance selectable marker or an empty vector control (Invitrogen, Carlsbad, CA, USA). For immunoblotting, whole-cell extracts were prepared in radioimmunoprecipitation assay (RIPA) lysis buffer (20 mm Tris-HCl, pH 7.5; 150 mm NaCl; 0.5% DOC, 1% NP-40 and 0.1% SDS). For cytoplasmic and nuclear fractionation, cells were first pelleted and resuspended in Buffer A (10 mm HEPES, pH 7.9; 1.5 mm MgCl2; 10 mm KCl). After 10 min of incubation on ice, lysates were pelleted and supernatant was collected as the cytoplasmic extract. The pellet was washed two times with Buffer A and resuspended in Buffer C (20 mm HEPES, pH 7.9; 25% glycerol; 420  mm NaCl; 1.5 mm MgCl_2_ and 0.2 mm EDTA). Tubes were incubated for 20 min on ice and then centrifuged to clear the nuclear extract. All lysis buffers were supplemented with protease and phosphatase inhibitors (2  mm Na3VO4; 1 mm NaF; 2 mm β-glycerolphosphate; 0.2  mm PMSF; 0.5  mm DTT and Complete protease inhibitor cocktail (Roche Diagnostics, Laval, QC, Canada)). Protein concentrations were determined by the BCA Protein Assay (Pierce, Rockford, IL, USA) according to the manufacturer's recommendations. Immunoblot analysis was performed on cell-equivalent lysates subjected to sodium dodecyl sulfate-polyacrylamide gel electrophoresis and electrophoretic transfer to polyvinylidene difluoride (Bio-Rad Laboratories, Mississauga, ON, Canada). Membranes were probed with anti-ELF3 (1:1000 dilution, Abcam anti-ESE1 antibody [EPESER1] (ab133621), Toronto, ON, Canada) and anti-Histone H3 (1:2000 dilution, #9715, Cell Signaling Technology, Danvers, MA, USA). For immunofluorescence analysis of 2-D monolayer cultures, cells were fixed in paraformaldehyde for 10 min Cells were then permeabilized using 0.05% Triton in PBS for 8 min After washing with 0.25% Tween in PBS, PBS containing 1% BSA was added to cells for 30  min to block non-specific interactions. Subsequently, anti-ELF3 (1:200, Sigma-Aldrich (HPA003479)) was added for 16 h at 4 degrees Celsius in 1% BSA in PBS. After washing with PBS, secondary antibodies were added for 30 min in 1% BSA in PBS at room temperature. Staining of filamentous actin was performed by adding rhodamine-conjugated phalloidin (1:400, Invitrogen, Burlington, ON, Canada). Coverslips were mounted in Fluoroshield Mounting Medium with DAPI to stain DNA (Abcam, Toronto, ON, Canada). Cell images were acquired with a Zeiss Colibri fluorescence microscope, AxioCam MRm camera and AxioVision Rel. 4.8 software (Carl Zeiss Canada Ltd., Toronto, ON, Canada). Cell viability of shRNA transfected HCC827 cells and isogenic controls was quantified calorimetrically using MTT reagent daily for 5 days. Cells were incubated for 4 h with MTT at 37 degrees Celcius before the reaction was ended by the addition of 20% SDS. Plates were left at room temperature overnight before scanning. Cell viability of A549 and H2030 cells was quantified calorimetrically by Alamar Blue 48 h post treatment with non-targeting control or pooled ELF3-targeting siRNAs. Cell proliferation was quantified using the BD PharmingenTM Apoptosis, DNA Damage and Cell Proliferation Kit (BD Bioscience, Mississauga, ON, Canada). Cells were incubated with BrdU for 8–24 h, and processed according to the manufacturer's instructions. In brief, cells were fixed, permeabilized, and treated with DNase I before staining with PerCP-CyTM5.5 Mouse Anti-BrdU antibody and DAPI (1 μg/ml). Cells were analyzed using the BD FACS CantoTM II cell analyzer (BD Bioscience). Experiments were repeated three times. Cell proliferation was also measured using the IncuCyte Live Cell Analysis system (Essen BioScience, Ann Arbor, MI, USA). For each cell line, a mask was created at the first measurement to determine average cell size, and applied to images taken every 2 h for 7 days to count the number of cells at each time point. Measurements were taken in technical sextet, and transformed standard deviation is show for a representative biological triplicate. Measurements of cell number at each time point were normalized to starting confluence of, as well as to plateau of growth achieved after 5–6 days (representing 100% confluence). For colony formation, single cell suspensions were prepared in growth media supplemented with 20% fetal bovine serum, and 0.3% low-melting point agarose (Invitrogen, Carlsbad, CA, USA). One milliliter of cell suspension was plated onto an equal volume of supplemented media with a 0.5% low-melting point agarose concentration. Each cell line was seeded in triplicate in 12-well plates and cultured for 14–21 days at 37˚C. Experiments were repeated three times. For HBEC-KT colony formation assays, 1000 viable cells were suspended and plated in 0.37% agar in Opti-MEM medium supplemented with 20% fetal bovine serum and 50 μg/mL bovine pituitary extract with 5 ng/mL EGF in triplicate 12-well plates, and were layered over a 0.50% agar base in the same medium as the one used for suspending the cells26. Cell apoptosis was quantified using the BD PharmingenTM Annexin V Apoptosis Detection Kit I according to the manufacturer’s instructions (BD Bioscience). Cells were grown in complete or serum free media for 72  h prior to cell processing, staining, and analysis by flow cytometry on the BD FACS CantoTM II cell analyzer (BD Bioscience). Live cells that were adherent at time of collection were used as a gating control.

### Xenograft tumor growth

All animal protocols complied with relevant ethical regulations and were approved by the Animal Care Committee of the University of British Columbia (Vancouver, British Columbia, Canada). ELF3 knockdown cell lines and controls were subcutaneously injected (A549: 2.5 × 10^6^ cells per site; HCC827: 5 × 10^6^ cells per site) into the left and right flanks of 6–8 week old NOD-SCID mice. Tumor volume was measured several times weekly until a total tumor burden of 1500 mm^3^ was achieved or tumors became ulcerated, at which point mice were euthanized. ELF3 overexpression cell lines and controls were subcutaneously injected (A549: 1.0 × 10^6^ cells per site) into the left and right flanks of 6–8 week old NRG mice. Tumor volume was measured several times weekly until a total tumor burden of 1500 mm^3^ was achieved or tumors became ulcerated, at which point mice were euthanized. For shELF3.cl-lacZ and shELF3.cl-OE xenografts (injected at 1.5 × 10^6^ cells per site), the slow growth rate resulted in a study endpoint whereby tumors reached a volume from which RNA could be extracted. DNA from FFPE xenografts was extracted using the Biostic FFPE Tissue DNA Isolation Kit (MO BIO Laboratories, Inc., Carlsbad, CA, USA) and cleaned up using the MiniElute Reaction Cleanup Kit (Qiagen, Hilden, Germany). DNA from cell line input was extracted using the DNeasy Blood & Tissue Kit (Qiagen, Hilden, Germany). DNA copies of lentiviral vector were quantified using Taqman Copy Number Assays, with primers designed to amplify the puromycin resistance cassette and calibrated against TFRC: Hs02677106_cn (diploid in HCC827); diploid reference assay for all samples was SFTPB: Hs01649948_cn. Copy number was assessed using CopyCaller Sofware v2.0 (Applied Biosystems, Carlsbad, CA, USA). After tissue homogenization, RNA was extracted according to standard Trizol protocols. Relative human cell-specific expression of *ELF3* (Hs00963882_g1 – no cross reactivity with mouse) and *NOTCH3* (Hs01128537_m1 – no cross reactivity with mouse) was quantified by qRT-PCR (TaqMan—Applied Biosystems) using mouse Actb (Mm04394036_g1) and human Actb (Hs99999903_m1) as endogenous controls. Whole-cell extracts were prepared in RIPA lysis buffer (20 mm Tris-HCl, pH 7.5; 150 mm NaCl; 0.5% DOC, 1% NP-40 and 0.1% SDS) and supplemented with protease and phosphatase inhibitors (2 mm Na_3_VO_4_; 1 mm NaF; 2 mm β-glycerolphosphate; 0.2 mm PMSF; 0.5 mm DTT and Complete protease inhibitor cocktail (Roche Diagnostics, Laval, QC, Canada)). All TaqMan assays were read in a 7500 Fast Real-Time PCR System (Applied Biosystems).

### PPI network and pathway analysis

For physical PPIs, we obtained 189 interacting partners of ELF3 and an additional 2526 PPIs among them (experimentally detected or computationally predicted interactions) from IID (ver. 4_2017; http://ophid.utoronto.ca/iid)^28^. Next, we annotated these PPIs with differential gene expression and differential gene co-expression data to construct tissue-specific and NSCLC-subtype-specific ELF3 interaction networks for further analyses. To investigate differential gene co-expression networks, gene expression data (HG-U133plus2 and HG-U133a chips) was downloaded from Gene Expression Omnibus (GEO, https://www.ncbi.nlm.nih.gov/geo/) and analyzed in batches, described below. Data were normalized using MAS5 method using Bioconductor package (Affy package version 1.48.0) available in R 3.2.3. For the pan-cancer analysis, data sets comprising samples from more than one tissue were separated into tissue-specific subsets. These data covered 1858 tumor (T) and 1026 non-malignant (N) treatment-naive patient samples across 13 tissue sites, including lung (*n* = 188 N, *n* = 510 T); breast (*n* = 86 N, *n* = 93 T); cervix (*n* = 30 N, *n* = 52 T); colon (*n* = 117 N, *n* = 345 T; endometrium (*n* = 21 N, *n* = 58 T; esophagus (*n* = 132 N, *n* = 159 T); kidney (*n* = 102 N, *n* = 181 T; liver (*n* = 20 N, *n* = 20 T); mouth (*n* = 67 N, *n* = 70 T); ovary (*n* = 49 N, *n* = 71 T); pancreas (*n* = 61 N, *n* = 73 T); prostate (*n* = 96 N, *n* = 143 T); thyroid (*n* = 57 N, *n* = 84 T). Lung subtype-specific analysis consisted of adenocarcinoma (*n* = 166 N, *n* = 398 T); squamous cell carcinoma (*n* = 35 N, *n* = 71 T); and large cell carcinoma (*n* = 33 N, *n* = 41 T).

Pan-cancer: GSE19383, GSE26910, GSE3744, GSE5764, GSE20437, GSE5462, GSE6883, GSE9574, GSE9750, GSE20916, GSE8671, GSE41258, GSE5364, GSE11024, GSE14762, GSE21816, GSE7023, GSE8271, GSE6280, GSE6344, GSE781, GSE29721, GSE14520, GSE31908, GSE14407, GSE15578, GSE18520, GSE36668, GSE38666, GSE15471, GSE16515, GSE22780, GSE17951, GSE32448, GSE32982, GSE3325, GSE6956, GSE29265, GSE3467, GSE3678, GSE6004, GSE27155, GSE17025, GSE20347, GSE23400, GSE29001, GSE30784, GSE31056, GSE33426, GSE38129, GSE53757, GSE64985, GSE7305, GSE7307, GSE7803.

Non-small cell lung cancer: GSE31210, GSE10245, GSE19188, GSE28571, GSE31908, GSE7670, GSE10072, GSE5364.

Next, for each pair of N and T samples, we calculated a Pearson Correlation Coefficient to generate a gene co-expression matrix across samples in N (i.e., *ρ*_*N*_) and across samples in T (i.e., *ρ*_*T*_). In the pan-cancer analysis, pairs of N and T samples were grouped by tissue. In the lung cancer subtype analysis, pairs of N and T samples were grouped by histological subtype. In the lung adenocarcinoma analysis, we restructured samples into 11 pairs of sample sets, each containing at least 20 T and 20 N samples. For each analysis we calculated differential gene co-expression matrix for each N and T pair:

1 $$Diff_{(N,T)} = \left| {\rho _N} \right. - \left. {\rho _T} \right|$$

We used the top 1% of values in the *Diff*_(*N,T*)_ matrix and overlaid it on the physical PPI network around ELF3 to define altered PPIs in cancer for each tissue. Next, we separated annotated PPIs into those lost in cancer ($\rho _N\, > \, \rho _T$) and those gained in cancer ($\rho _T\, > \, \rho _N$).

*Differential gene expression network*: we used level 3 mRNA expression data of 498 LUAD patient samples from TCGA. We removed genes with more than 10% missing values. For 15,448 remaining genes, we replaced missing values with average of available values across all samples of each gene. In all, 125 samples in the top quartile of ELF3 mRNA expression distribution were used as ELF3^high^ and 125 samples in the bottom quartile of this distribution were used as ELF3^low^ samples. ELF3 expression of all samples in ELF3^high^ set was at least two times of its expression in all samples in ELF3^low^ set. Next, we stratified each group of samples into two groups: (1) KRAS^mut^ samples (41 ELF3^high^ and 31 ELF3^low^ samples) and KRAS^wt^ samples (84 ELF3^high^ and 94 ELF3^low^ samples).

For each of the KRAS^mut^ and KRAS^wt^ groups, we calculated differential mRNA expression between ELF3^high^ and ELF3^low^ samples (two-tailed *t* test). After correcting raw *p* values for multiple hypothesis testing using Benjamini–Hochberg (false discovery rate) method we used genes with corrected *p* value < 0.05 and fold change of at least 1.2 as differentially expressed genes to annotate proteins in the first level network of ELF3.

For isogenic cell lines, *w*e used mRNA expression of three replicates of shELF3 A549 cell line and three control samples (explained above). Owing to low analysis power which is a result of small number of samples, we did not use standard gene expression analysis on cell lines, rather, we used difference of geometric mean and raw *p* value across genes between shELF3 and control lines to further validate our TCGA KRASm^mut^ network analysis.

In order to summarize consistency and rank proteins based on their alterations across different conditions we defined three support score components as the following: a protein has full support in a data set if its alteration passes the defined threshold; a protein has partial support in a data set if it satisfies two conditions: (a) it has full support through another data set, and (b) its alteration level in this data set is slightly less than the defined threshold (for example, TIMP3 is partially supported in TCGA-LUAD-KRAS^wt^ set, since it has full support from GEO and TCGA-LUAD-KRAS^mut^ (further supported through cell lines), and its differential expression *q* value is slightly above 0.05 (between 0.05 and 0.06) in TCGA-LUAD-KRAS^wt^), and a gene has no support if it is present neither fully nor partially supported.

Full support from each data set for a protein increases its support score by 2 points, partial support increases its support score by 1 point and no support has value 0. The final support score for each protein is calculated by adding up these three score components.

For pathway analysis, we used pathDIP (version 2.5; http://ophid.utoronto.ca/pathDIP)^29^, extended pathways based on experimental and computational PPIs at cutoff association score of 0.95, to perform pathway enrichment analysis across the above three pairs of differential networks (GEO N vs T, TCGA KRAS^mut^ ELF3^low^ vs ELF3^high^, and TCGA KRAS^wt^ ELF3^low^ vs ELF3^high^). Next, we used term enrichment analysis tool in pathDIP to further summarize enriched pathways (pathways with corrected *q* value < 0.05). The results are presented in Supplementary Fig. 12, and additional relevant details are available in Supplementary Data 1.

### Survival analysis

Meta data for Kaplan–Meier survival analysis were obtained from http://kmplot.com/analysis/index.php?p=service&cancer=lung^40^. Samples were ranked by *ELF3* expression and survival curves of the top and bottom expression tertiles were compared by log-rank analysis.

### Reporting summary

Further information on research design is available in the Nature Research Reporting Summary linked to this article.

## Supplementary information

Supplementary Information

Description of Additional Supplementary Files

Supplementary Data 1

Supplementary Data 2

Reporting Summary

## Data Availability

Gene expression data from paired LUAD and NM tissues can be accessed in the GEO repository under the accession code GSE75037. All data generated in this study, including LUAD and NM Affymetrix copy number SNP6.0 array data, LUAD and NM Illumina HumanMethylation27 data, and A549 Affymetrix expression microarray data have been deposited in the GEO repository as a Super Series under the accession code GSE137481. Raw LUSC and NM RNA-sequencing data are available under the BioProject PRJNA563664. The gene expression profiles used to construct pan-tissue ELF3 protein–protein interaction networks are available in the GEO repository under the accession codes GSE19383, GSE26910, GSE3744, GSE5764, GSE20437, GSE5462, GSE6883, GSE9574, GSE9750, GSE20916, GSE8671, GSE41258, GSE5364, GSE11024, GSE14762, GSE21816, GSE7023, GSE8271, GSE6280, GSE6344, GSE781, GSE29721, GSE14520, GSE31908, GSE14407, GSE15578, GSE18520, GSE36668, GSE38666, GSE15471, GSE16515, GSE22780, GSE17951, GSE32448, GSE32982, GSE3325, GSE6956, GSE29265, GSE3467, GSE3678, GSE6004, GSE27155, GSE17025, GSE20347, GSE23400, GSE29001, GSE30784, GSE31056, GSE33426, GSE38129, GSE53757, GSE64985, GSE7305, GSE7307, GSE7803; gene expression profiles used to construct ELF3 protein–protein interaction networks in non-small cell lung cancer are available under the accession codes GSE31210, GSE10245, GSE19188, GSE28571, GSE31908, GSE7670, GSE10072, GSE5364 (https://www.ncbi.nlm.nih.gov/geo/). Other data sets referenced during the study are available from the TCGA Data Portal [https://tcga-data.nci.nih.gov/tcga/tcgaDownload.jsp], deposited in GEO under the accession numbers GSE3141 and GSE8894, and the Kaplan–Meier Plotter Lung Cancer webpage (http://kmplot.com/analysis/index.php?p=service&cancer=lung). All the other data supporting the findings of this study are available within the article and its supplementary information files and from the corresponding author upon reasonable request. A reporting summary for this article is available as a Supplementary Information file.

## Source data

Source Data
