# Supplementary material for: Epithelial tumor suppressor ELF3 is a lineage-specific amplified oncogene in lung adenocarcinoma
Source: Nat Commun. 2019 Nov 28;10:5438. doi: 10.1038/s41467-019-13295-y (PMC6882813; doi:10.1038/s41467-019-13295-y)
Supplement: Supplementary file 1 — Supplementary Information [file 41467_2019_13295_MOESM1_ESM.pdf]

Epithelial Tumour Suppressor ELF3 is a Lineage-Specific Amplified Oncogene in Lung Adenocarcinoma

Enfield *et al*.

Supplementary Information

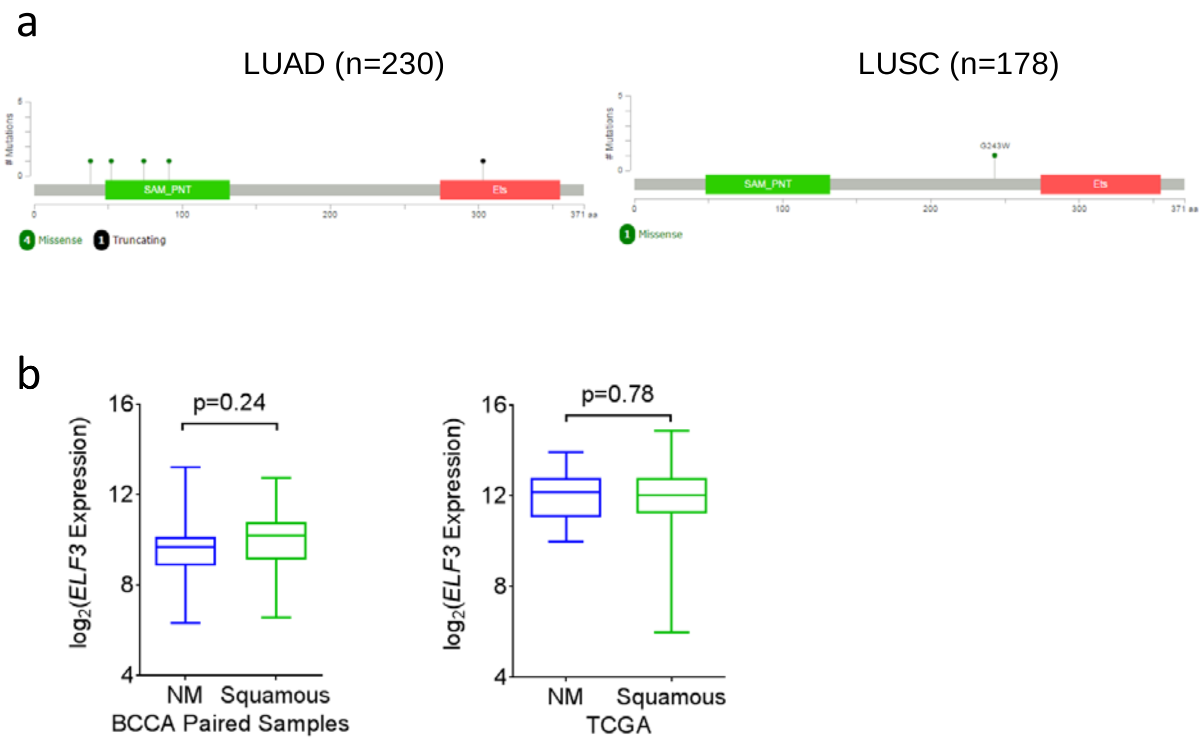

Supplementary Fig. 1. Characterization of ELF3 in lung adenocarcinoma and squamous cell carcinoma. (a) ELF3 mutations detected in previous studies of LUAD[23] (2.2%) and LUSC[56] (0.6%). Figure generated in cBioPortal[52,53]. (b) Comparison of *ELF3* expression between non-malignant lung (NM, blue) and lung squamous cell carcinoma (LUSC, green) in the BCCA dataset (n=58, Wilcoxon sign-rank test), and the TCGA dataset (n=555, Mann Whitney U test, center line represents the median, box bounds indicate the 25th and 75th percentiles, and whiskers extend from minimum to maximum).

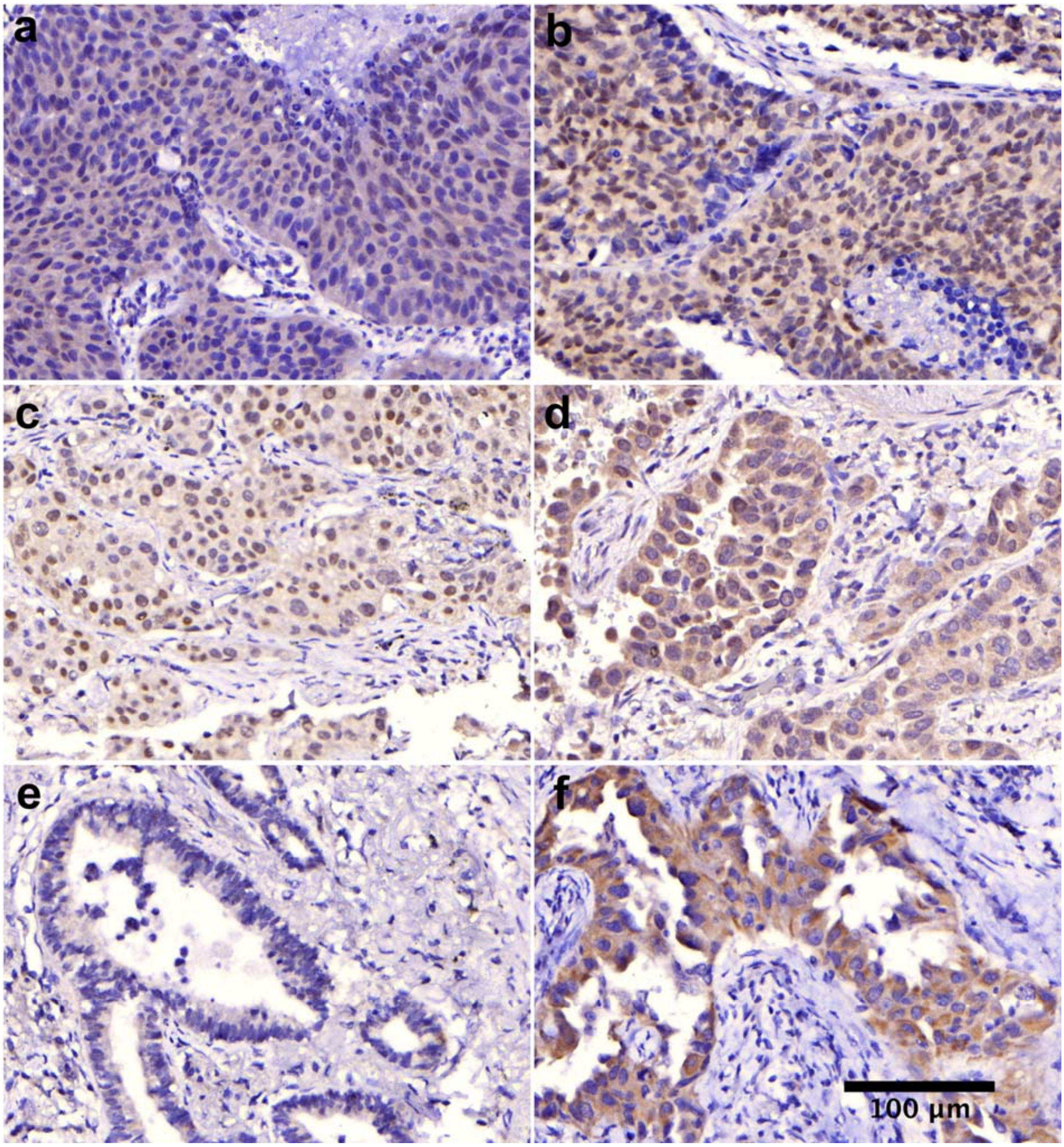

Supplementary Fig. 2. Immunohistochemistry staining of ELF3 in non-small cell lung cancer. Representative ELF3 immunohistochemistry images from a tissue microarray of LUSC (a), and LUAD (b-f). One example of LUAD with no detectable ELF3 expression is shown in panel e. Scale bar = 100µm.

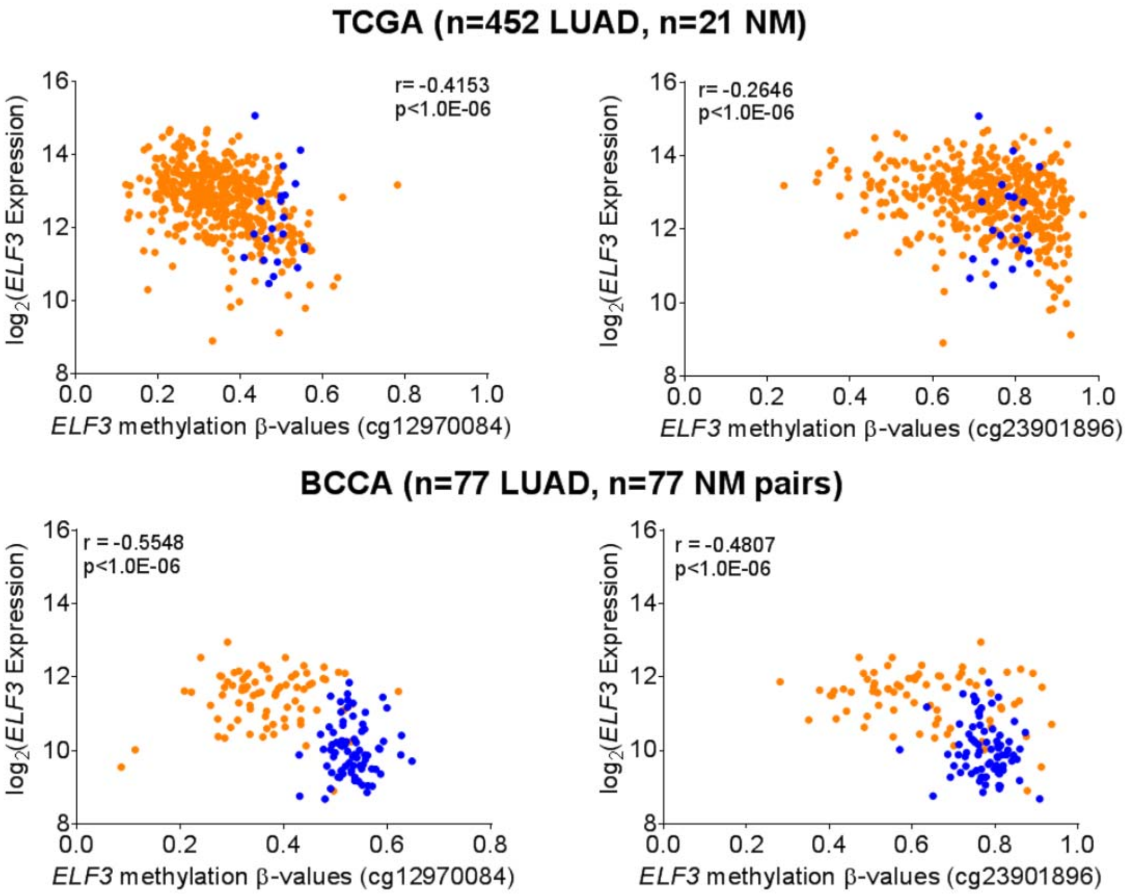

Supplementary Fig. 3. Inverse correlation of *ELF3* promoter methylation with RNA-level expression. Spearman correlation of CpG probe beta values (cg12970084 and cg23901896) with *ELF3* expression in TCGA and BCCA multi-omics datasets. Blue dots=NM; orange dots=LUAD. Probe cg12970084 was most significantly associated with expression and was included in downstream analysis (TCGA: ρ=-0.415, $p<1.0\times10^{-6}$; BCCA: ρ=-0.5548, $p<1.0\times10^{-6}$).

TCGA-60 dataset (n=252 LUAD, 21 NM)

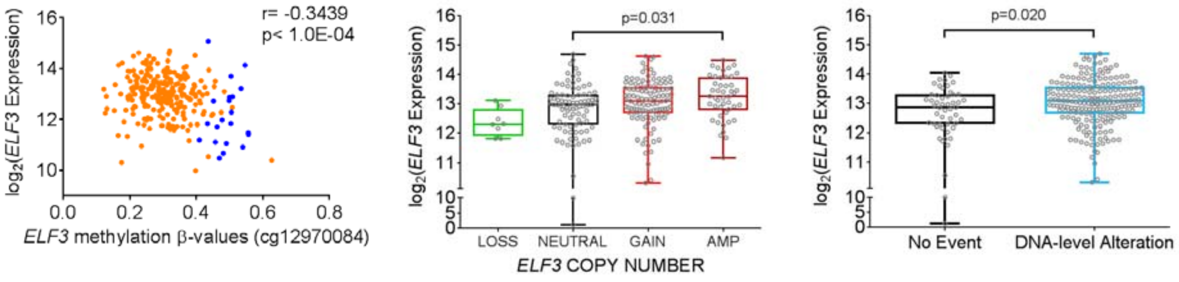

BCCA dataset (n=83 pairs)

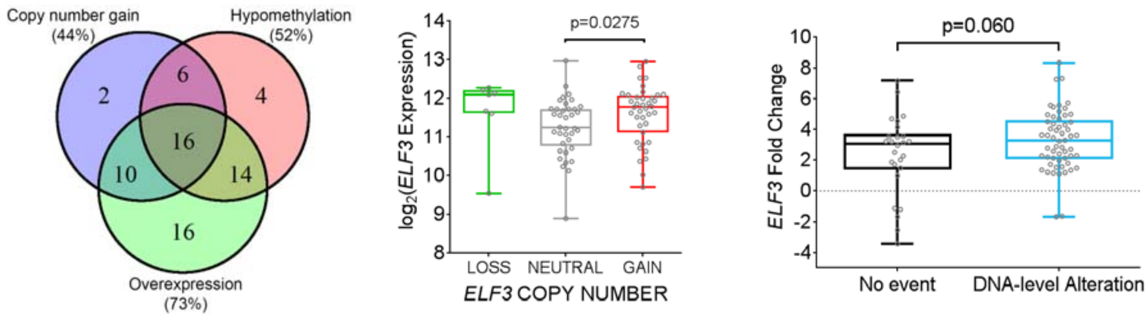

Supplementary Fig. 4. *ELF3* expression is associated with DNA-level locus alterations in the TCGA-60 and BCCA datasets.

TCGA-60 (TCGA cases with ≥60% tumour cellularity): (left panel) Scatter plot of *ELF3* promoter methylation (x-axis) and ELF3 expression (y-axis) across 252 LUAD (orange) and 21 non-malignant lung (blue) samples (ρ=-0.3439, p<1.0×10^{-4} by Spearman's correlation). (Middle panel) *ELF3* expression as a function of DNA copy number. (Right panel) Comparison of *ELF3* expression between tumours with DNA gain/amplification or promoter hypomethylation (blue outline) compared to those without (black outline). Mann-Whitney U test p-values are shown. BCCA: (left panel) Venn diagram of tumours with *ELF3* DNA copy number gain, promoter hypomethylation, or >2-fold overexpression. (Middle panel) *ELF3* expression as a function of DNA copy number. (Right panel) Comparison of *ELF3* expression between tumours with DNA gain/amplification or promoter hypomethylation (blue outline) compared to those without (black outline). Box and whiskers plots: center line represents the median, box bounds indicate the 25th and 75th percentiles, and whiskers extend from minimum to maximum. Mann-Whitney U test p-values are shown.

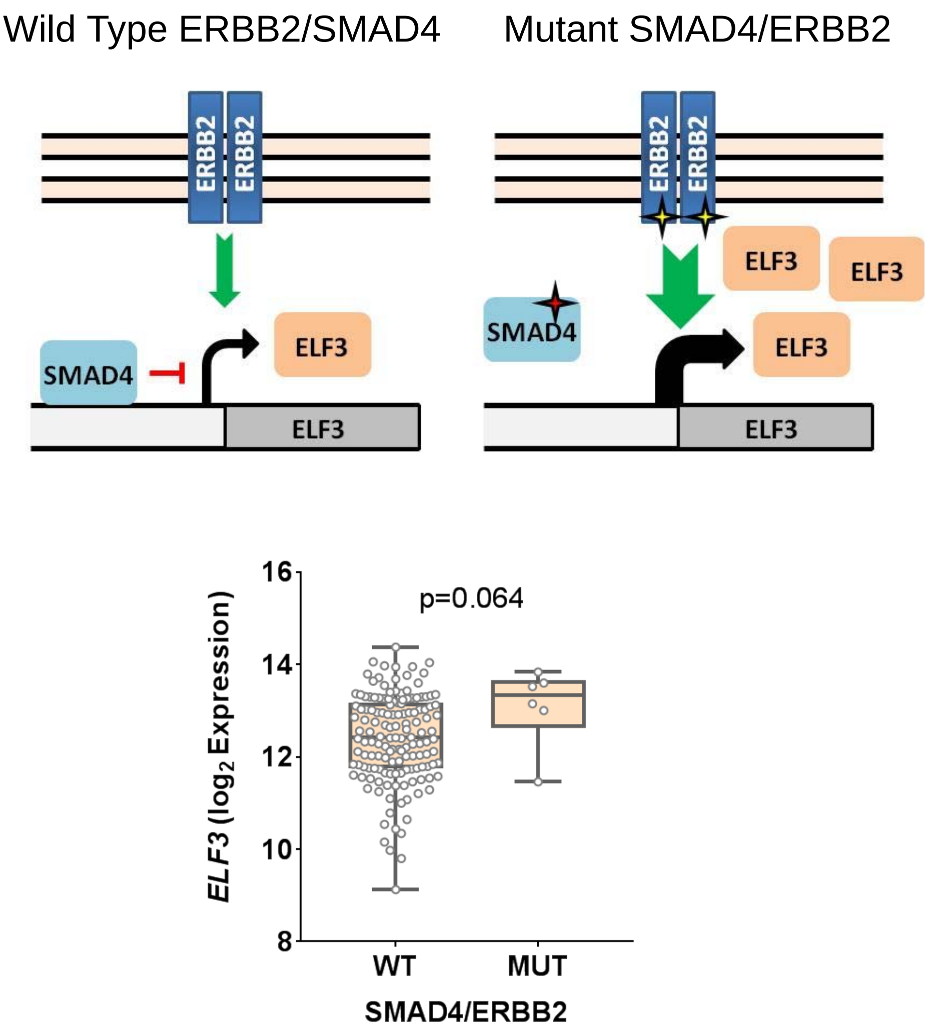

Supplementary Fig. 5. Mutations in SMAD4 and ERBB2 are associated with increased *ELF3* expression. Schematic indicating the ELF3-promoting function of ERBB2 signalling, and transcriptional repression of ELF3 by SMAD4[17]. Mutations leading to constitutive activation of ERBB2, as well as deleterious mutations in SMAD4 result in increased promotion of ELF3 expression. This effect is summarized in the box and whisker plots comparing TCGA LUAD harboring wild type or mutant SMAD4 or ERBB2 (Mann-Whitney U test, center line represents the median, box bounds indicate the 25th and 75th percentiles, and whiskers extend from minimum to maximum). Only tumours lacking *ELF3* locus alterations were considered.

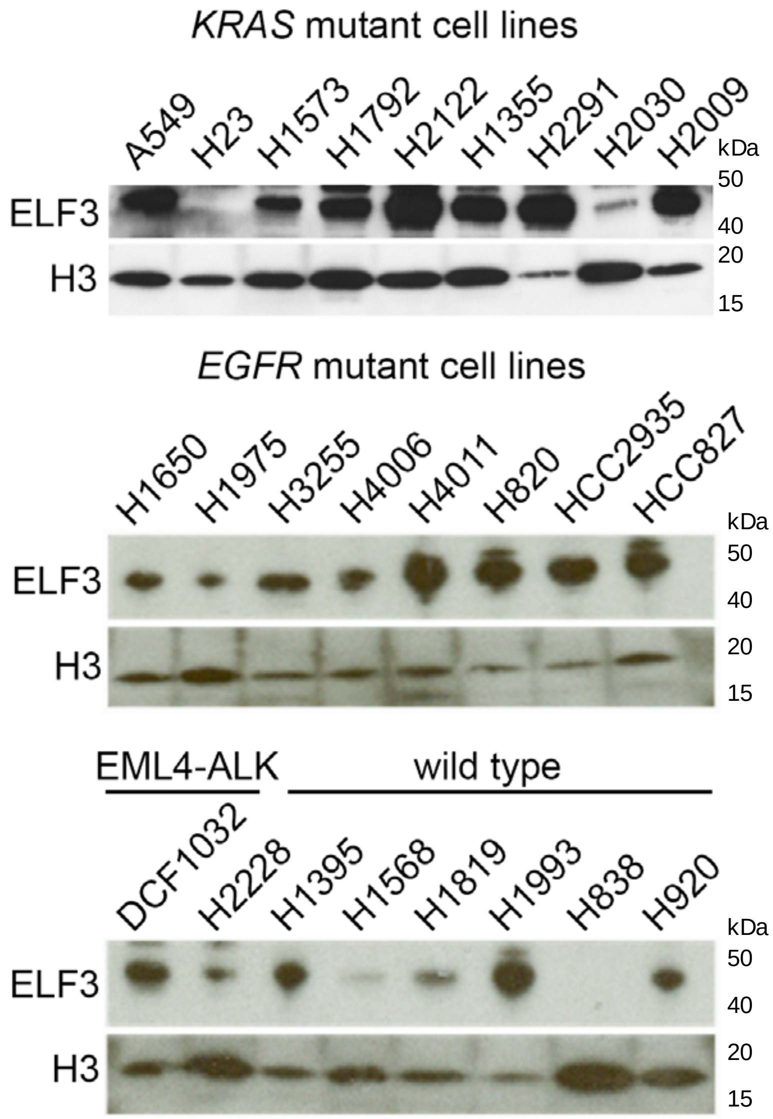

Supplementary Fig. 6. Investigation of ELF3 across molecular subtypes of lung adenocarcinoma. Immunoblot of ELF3 expression in a panel of KRAS mutant, EGFR mutant and EML4-ALK positive LUAD cell lines, as well as in LUAD cell lines lacking these alterations (wild type). Histone H3 was used as a loading control.

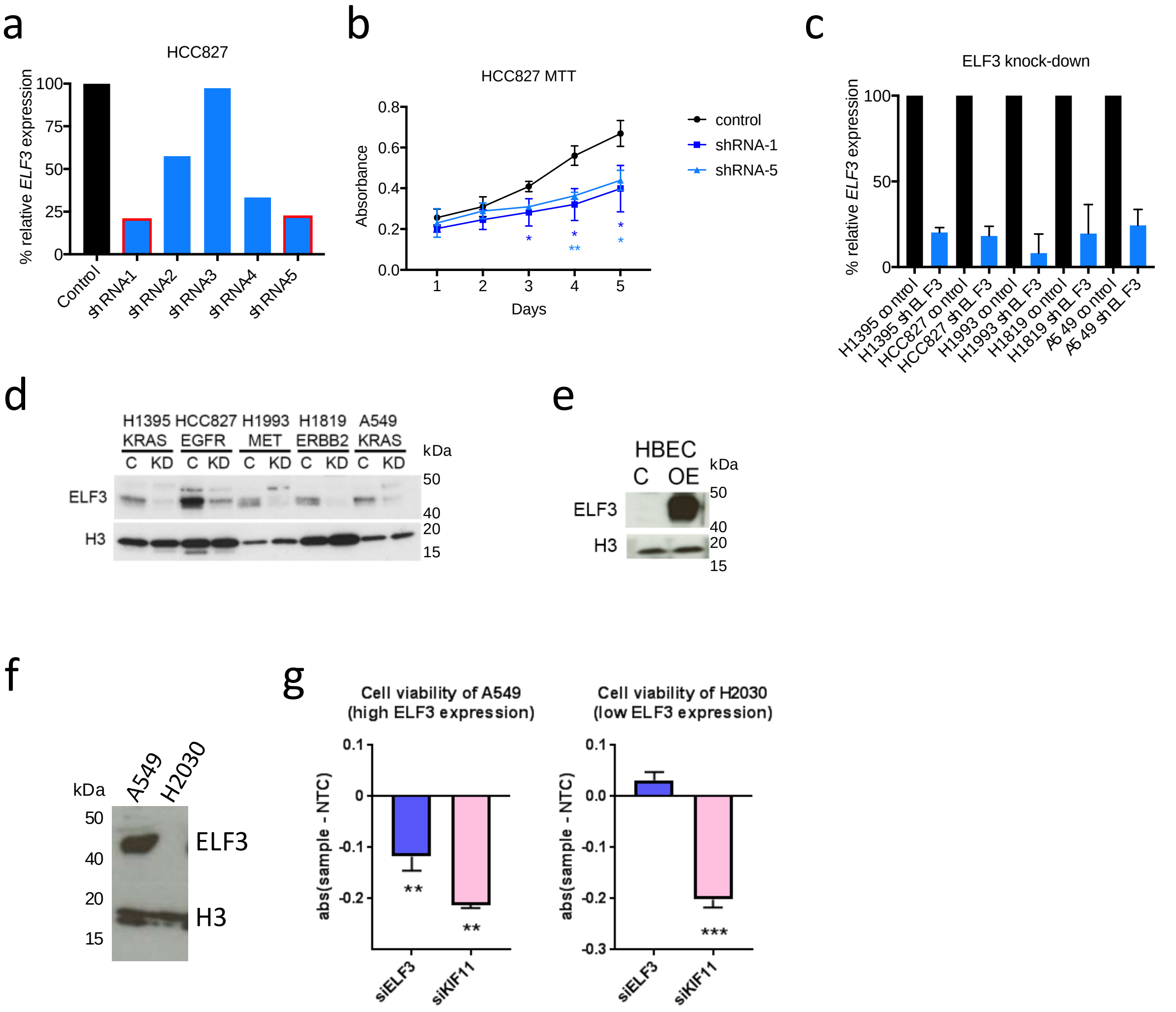

Supplementary Fig. 7. *In vitro* manipulation of ELF3 expression.

(a) qRT-PCR results indicating percent ELF3 knock-down achieved by 5 independent shRNAs in HCC827 cells (blue bars) compared to isogenic control (black bars). The two shRNAs with the greatest proportion of knock-down (shRNA-1 and shRNA-5) are highlighted in red. (b) Results from biological triplicate MTT assays comparing viability of HCC827 cells stably expressing shRNA-1 (royal blue line) or shRNA-5 (light blue line) compared to empty vector control (black line). (c) qRT-PCR results indicating percent *ELF3* knock-down (blue bars, mean + SEM) achieved in at least three biological replicate experiments compared to isogenic controls (black bars) for shRNA-1 or shRNA-5 in lung adenocarcinoma cell lines. (d) Corresponding immunoblot of ELF3 expression in lung adenocarcinoma cells lines transfected with control or shRNA knock-down (KD) vector. Histone H3 was used as a loading control. (e) Representative immunoblot of ELF3 expression in HBECs transfected with control or ELF3 overexpression (OE) vector. (f) Immunoblot of ELF3 expression in A549 ELF3-positive and H2030 ELF3-negative LUAD cell lines. (g) Cell viability of A549 and H2030 cells following treatment with ELF3 targeting siRNAs or positive control KIF11 targeting siRNAs relative to non-targeting controls. (b and g) Two-tailed paired Student's t-test p-values: *p<0.05, **p<0.01, ***p<0.001.

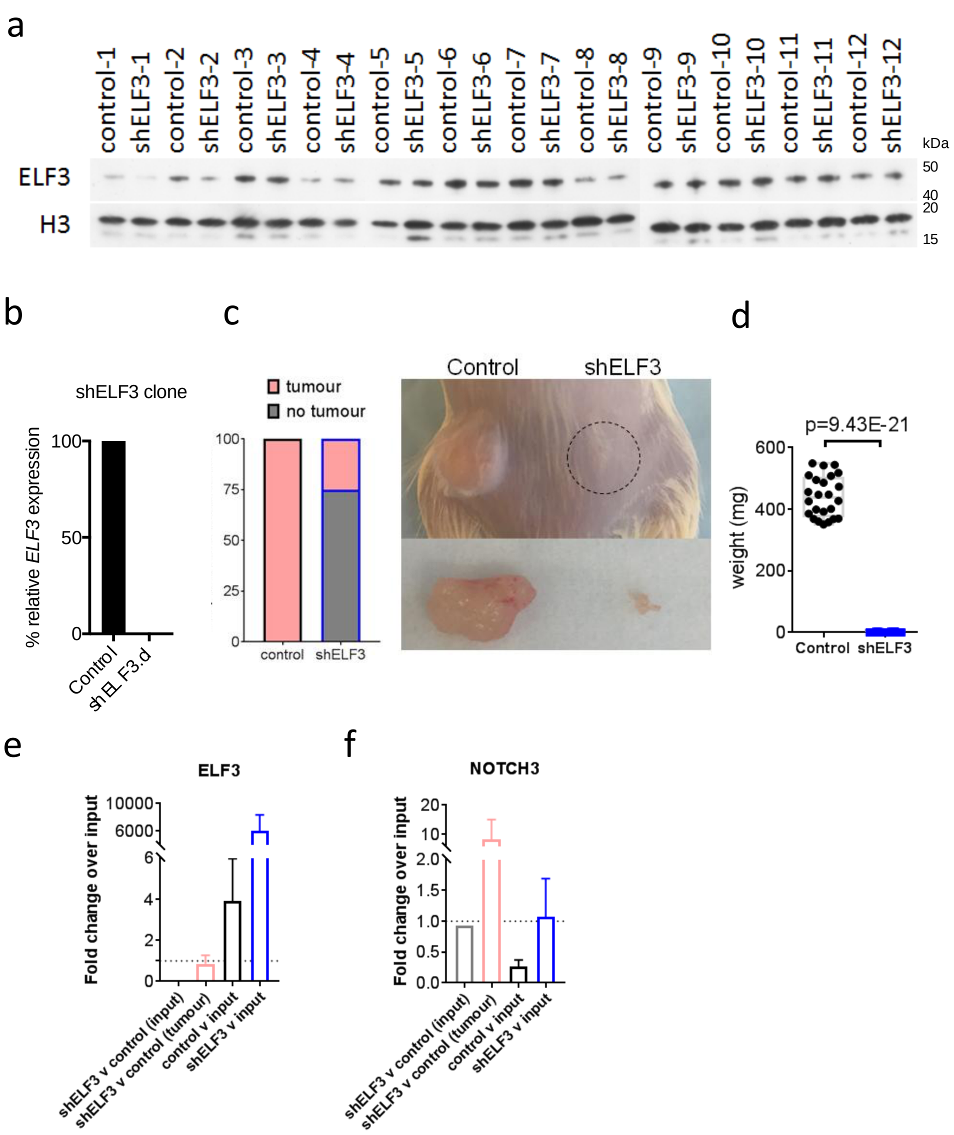

Supplementary Fig. 8. Effect of ELF3 inhibition of *in vivo* growth.
(a) Immunoblot of ELF3 expression in polyclonal control and shELF3 HCC827 xenografts collected at endpoint Day 42. Histone H3 was used as a loading control. (b) Proportion of successful xenograft development initiated by control and clonal shELF3 A549 cells. 2.5×10^{6} cells were injected per site (n=24 mice). Image indicates a small mass detected at the shELF3 injection site (6/24 mice). (c) Comparison of control and shELF3 xenograft weights at endpoint (two tailed paired Student's t-test, $p=9.43 \times 10^{-21}$, center line represents the median, box bounds indicate the 25th and 75th percentiles, and whiskers extend from minimum to maximum). Fold change (FC) in expression (RQ value) of (d) *ELF3* and (e) *NOTCH3* as determined by qPCR. Grey bars indicate relative expression in input cell line material (*ELF3* FC=0.00, *NOTCH3* FC=0.94); pink bars indicate relative expression in endpoint xenografts (*ELF3* FC=0.86, *NOTCH3* FC=8.4); black bars indicate expression of control xenografts with reference to input control cell line expression (*ELF3* FC=3.9, *NOTCH3* FC=0.26); blue bars indicate expression of shELF3 xenografts with reference to input shELF3 cell line expression (*ELF3* FC=6062, *NOTCH3* FC=1.1). Error bars represent SEM in all cases.

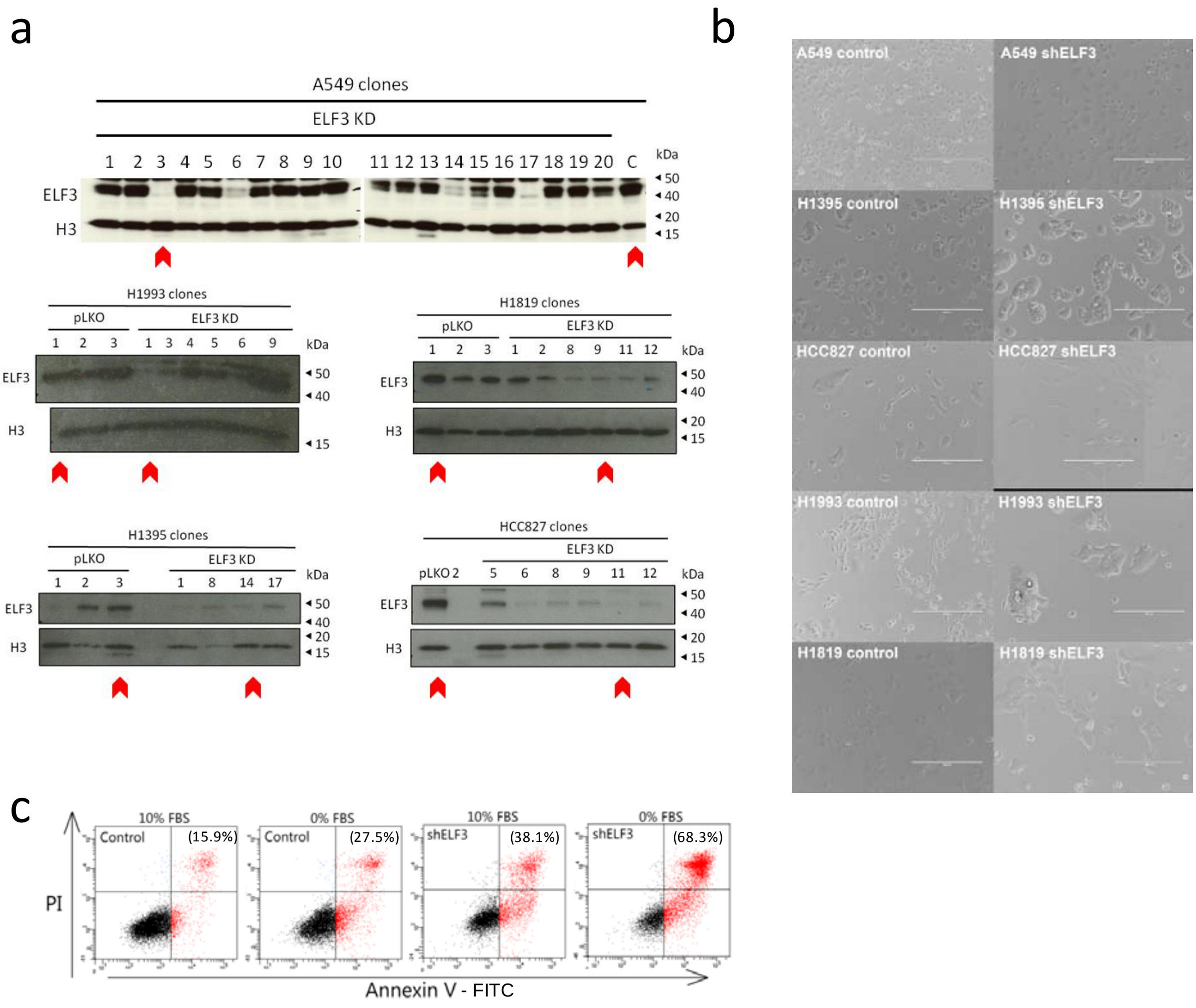

Supplementary Fig. 9. Clonal ELF3 inhibition results in morphological changes and decreased cell viability.

(a) Selection of clonal populations of control (pLKO) and shELF3/ELF3 KD cell lines. ELF3 expression was assessed by immunoblot with Histone H3 used as a loading control. Selected clones are indicated by a red arrow. (b) Representative images of the selected clonal populations (10x magnification, scale bar = 400µm). (c) Representative flow cytometry data. Cells were stained for Annexin V and PI following culture in complete (10% FBS) or depleted media (0% FBS). Annexin V-FITC positive cells are shown in red. Gates were generated based on live singlets.

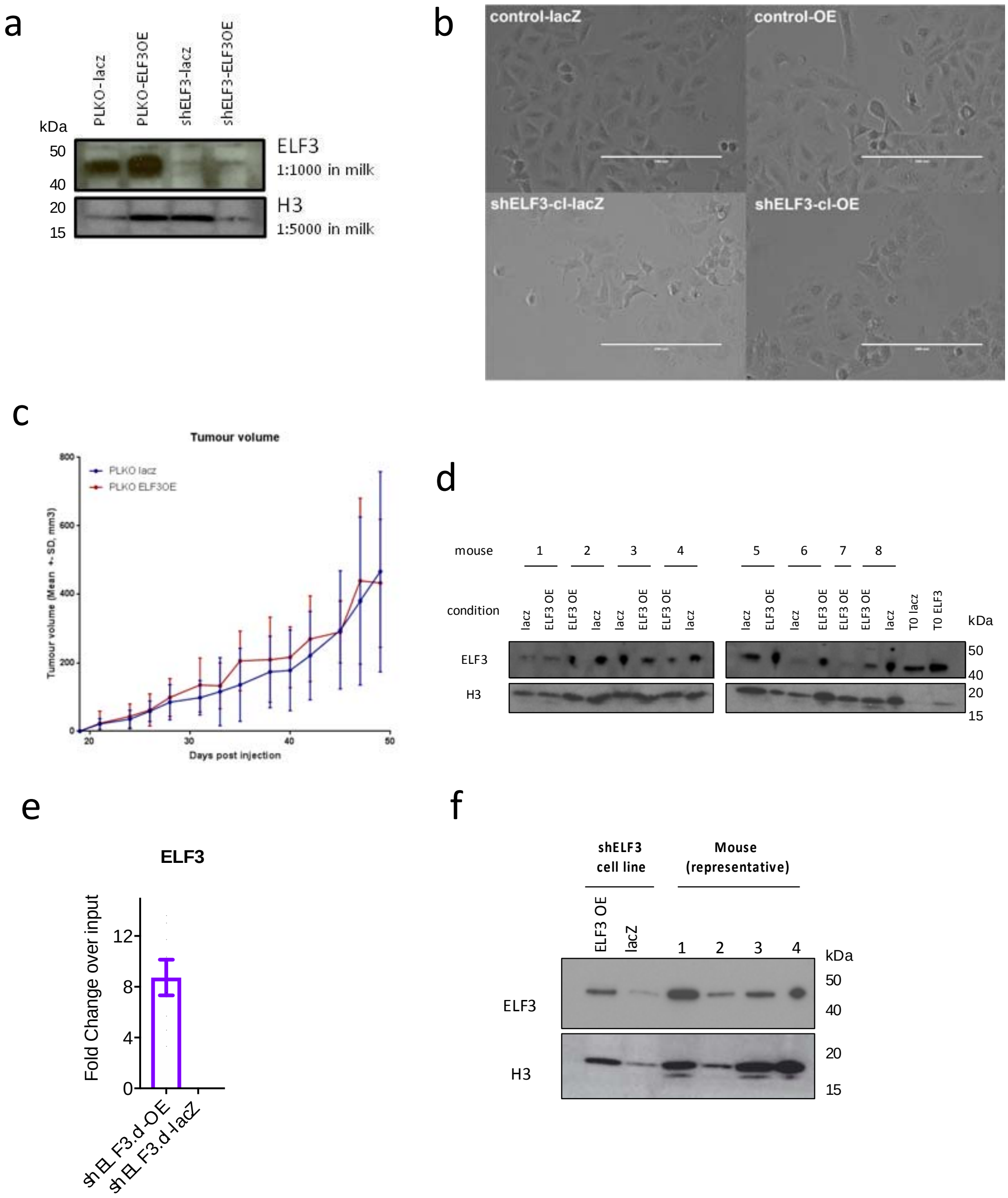

Supplementary Fig. 10. ELF3 expression rescue restores morphology and oncogenic phenotypes. (a) Immunoblot of ELF3 expression in clonal A549 cells (control and shELF3) following stable ELF3 overexpression. Histone H3 was used as a loading control. (b) Representative images of clonal A549 cells following ELF3 overexpression (20x magnification, scale bar = 200µm). (c) Tumour growth curve of control (PLKO) lacZ and control (PLKO) OE A549 cells implanted into NRG mice (n=8) (mean + SEM). (d) Immunoblot of ELF3 expression in control lacZ and control OE A549 xenografts collected at endpoint Day 54. Histone H3 was used as a loading control. (e) Fold change (FC) in expression (RQ value) of *ELF3* as determined by qPCR in clonal shELF3 cells (lacZ and OE). Purple bars indicate relative expression in shELF3.cl-OE endpoint xenografts compared to input cell line material, error bars indicate SEM. No shELF3.cl-lacZ tumours were present at endpoint. (f) Immunoblot of ELF3 expression in input shELF3.cl-lacZ and shELF3.cl-OE A549 cell lines at Day 0, and four representative shELF3.cl-OE xenografts collected at endpoint Day 54. Histone H3 was used as a loading control.

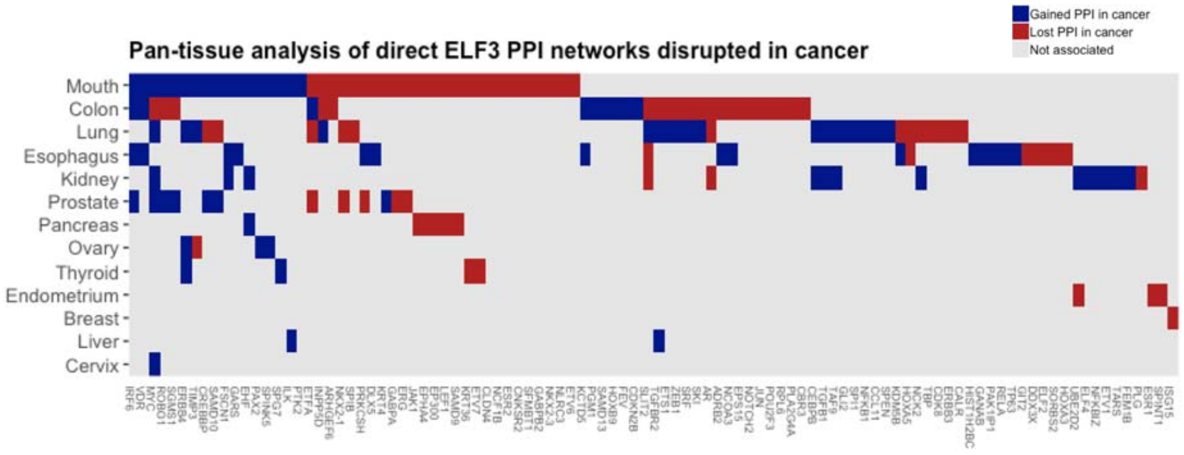

Supplementary Fig. 11. Pan-cancer analysis of altered direct ELF3 protein-protein interactions. Tissues are ordered vertically according to decreasing number of altered direct ELF3 PPIs in each cancer type. Disrupted PPIs were identified by comparing cancer profiles to non-malignant profiles in the same tissue types, with PPIs gained in cancer shown in red, and those lost in cancer shown in blue. Of the 189 direct ELF3 PPIs in IID, 101 were disrupted in at least one cancer type in this analysis. Lung cancer accounts for 30% of these altered PPIs, with 18 PPIs lost and 13 PPIs gained in cancer.

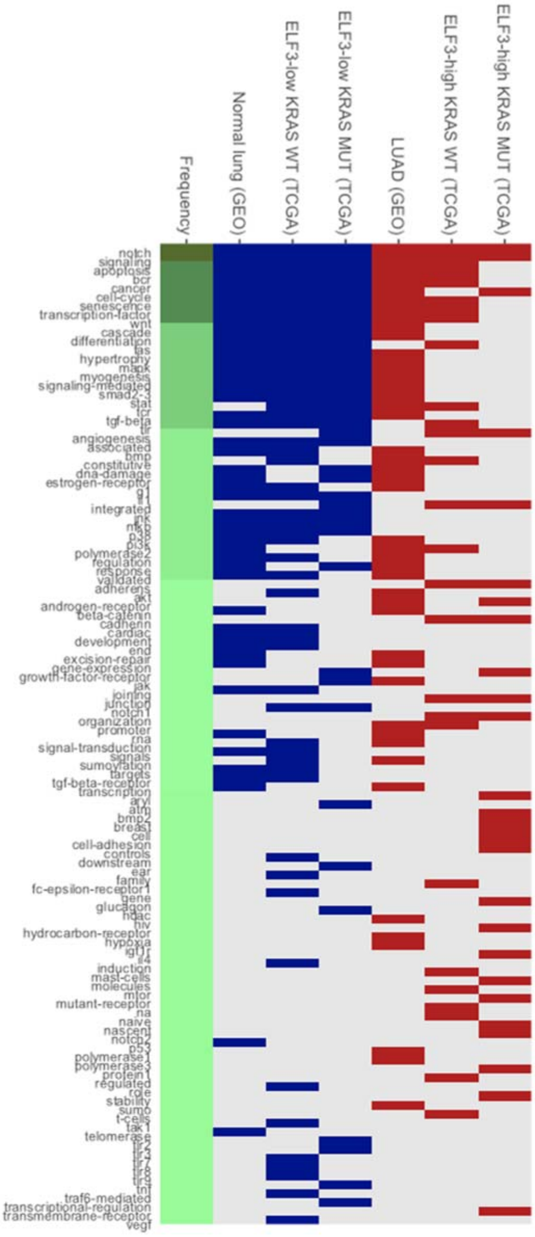

Supplementary Fig. 12. Most significantly enriched terms in condition-specific ELF3 protein-protein interaction networks.

Summary of pathway enrichment analysis of ELF3 condition-specific PPIs across six conditions: LUAD vs. non-malignant lung (GEO), ELF3-high vs. ELF3-low KRAS mutant (TCGA), ELF3-high vs. ELF3-low KRAS wildtype (TCGA). The plot shows keywords enriched in at least one of these six conditions, ordered by frequency from high (dark green) to low (light green). The full list of enriched terms and relevant corrected p-values are available in Supplementary Table 3. NOTCH is the only pathway that shows significant alterations in all conditions. Furthermore, while TCGA KRAS^{wt} ELF3^{low} expresses the maximum number of shared terms with non-malignant lung, TCGA KRAS^{mut} ELF3^{high} shows the least similarity to all other conditions and is most similar to TCGA KRAS^{wt} ELF3^{high}.

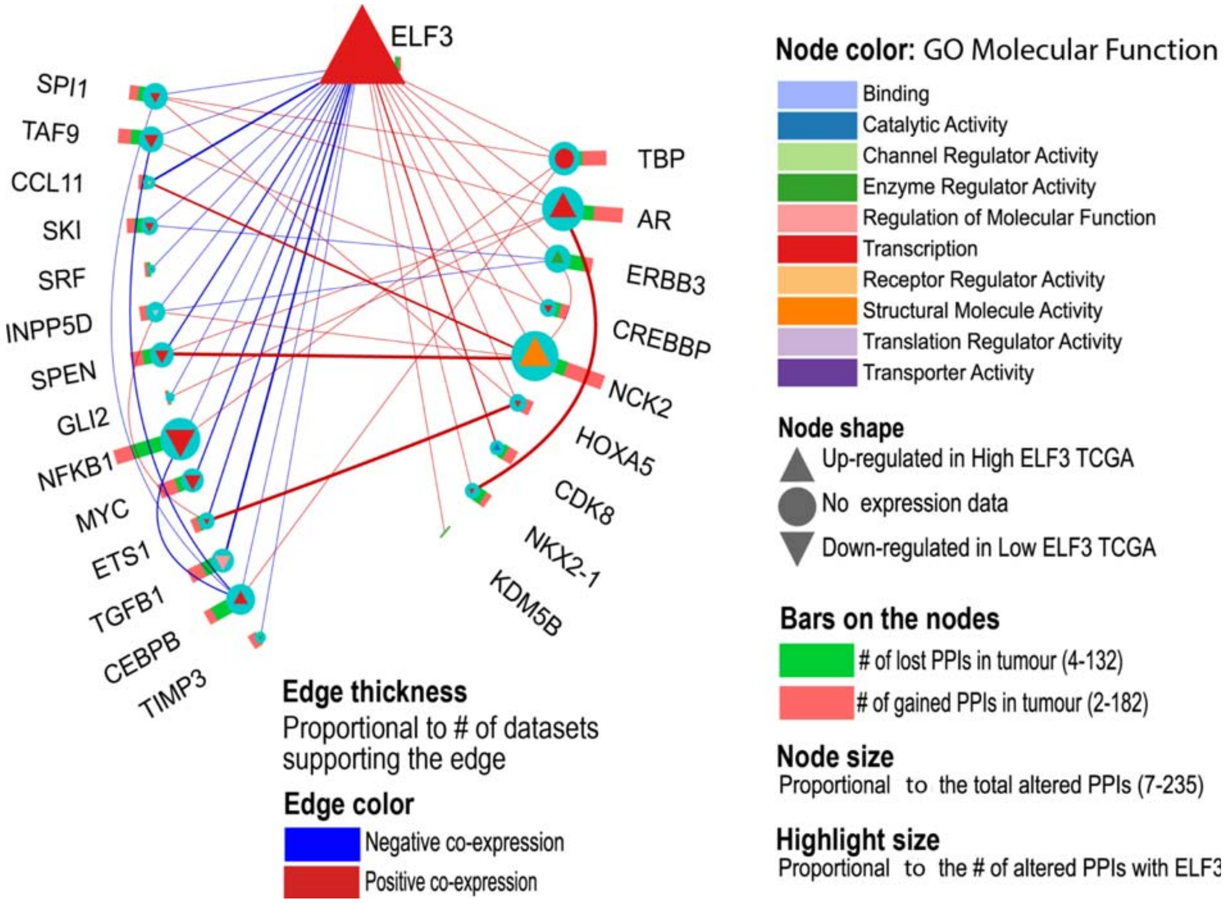

Supplementary Fig. 13. Altered ELF3 protein-protein interaction network in non-malignant lung vs. lung adenocarcinoma.

Of the 189 ELF3 PPIs in IID, the 23 shown here have highly altered co-expression with ELF3 in LUAD compared with non-malignant lung (GEO data). Considering GO functional annotations for these genes, transcription factor activity (13/23) is the most prevalent function among these differentially expressed genes. This network is the union of the altered network around ELF3 in LUAD after overlaying differential gene co-expression in 11 LUAD vs. non-malignant lung sample sets. The edge thickness represents how frequently they have appeared across these 11 networks. Additional validation using TCGA LUAD $ELF3^{high}$ vs. $ELF3^{low}$ samples shows down-regulation of lost PPI partners in $ELF3^{high}$ TCGA samples (blue edges, down triangles), and up-regulation of gained PPI partners in $ELF3^{high}$ samples (red edges, up triangles), suggesting that mRNA expression of normal-specific partners of ELF3 is higher in tumours with low ELF3 expression, compared with tumours with high ELF3 expression. However, CREBBP and HOXA5 on the right side and CEBPB on the left side show different behaviour from other proteins in this network, which could be due to the differences between non-malignant lung and LUAD PPI networks, independent of ELF3 levels. Node colour indicates GO molecular function, node shape indicates directionality of expression change in TCGA data, node size is proportional to the total number of altered PPIs, and node highlight size is proportional to the number of altered PPIs in the ELF3 network. Bars on the nodes indicate the number of lost (green bar) and gained (pink bar) PPIs in LUAD.

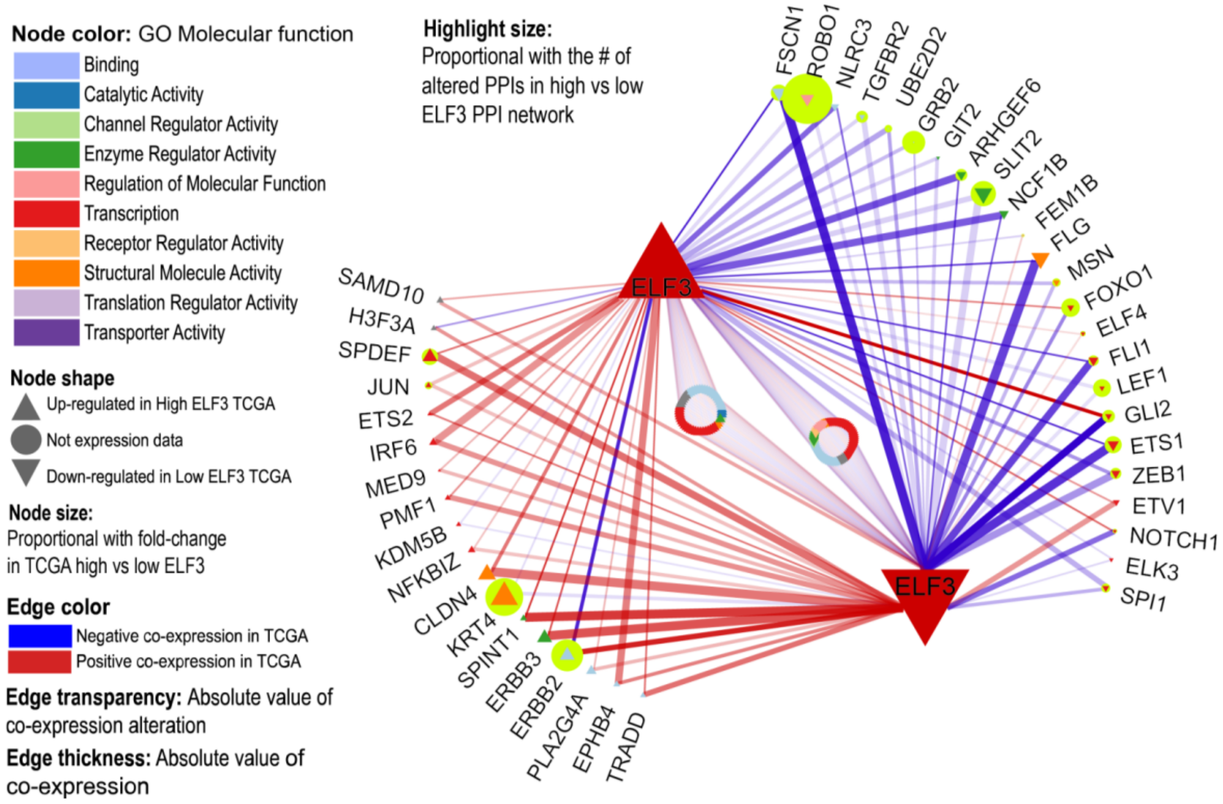

Supplementary Fig. 14. Differentially expressed ELF3 interacting partners in KRAS wild type lung adenocarcinoma.

The difference between mRNA expression of ELF3 PPIs was calculated in ELF3^{high} vs. ELF3^{low} samples with KRAS^{wt} (TCGA). Fourty-two out of 158 PPI partners of ELF3 that were available in the TCGA dataset were significantly de-regulated when ELF3 was over-expressed. Considering GO functional annotations for these genes (node colour), transcription activity (19/42), binding (7/42), and enzyme activity (6/42), are the most prevalent functions among these differentially expressed genes. Proteins at the left-side show significantly up-regulated (up-triangles) mRNAs when ELF3 is highly expressed with predominantly positive co-expression (red edges), and right-side proteins represent significantly down-regulated (down-triangles) mRNAs when ELF3 is highly expressed with predominantly negative co-expression (blue edges). Node shape indicates directionality of expression change in TCGA KRAS^{wt} data, node size is proportional to the total number of altered PPIs, and node highlight size is proportional to the number of altered PPIs in the ELF3 network. Circles in the centre indicate ELF3 partners whose differential expression was not statistically significant. Pathway analysis results show that while both groups of deregulated (up and down) genes are enriched with terms including (but not limited to) NOTCH, BCR, Apoptosis, Transcription, FAS, and TCR, there are terms which are different between up- vs. down-regulated genes. For example, cell-cycle, MAPK, myogensis, SMAD1-2, and STAT are among terms which are enriched only with down-regulated partners of ELF3 where ELF3 is highly expressed. Conversely, cadherin, adherens, and junction, are among terms which are enriched only with up-regulated partners of ELF3 where it is highly expressed (for details see Supplementary Table 3).

a

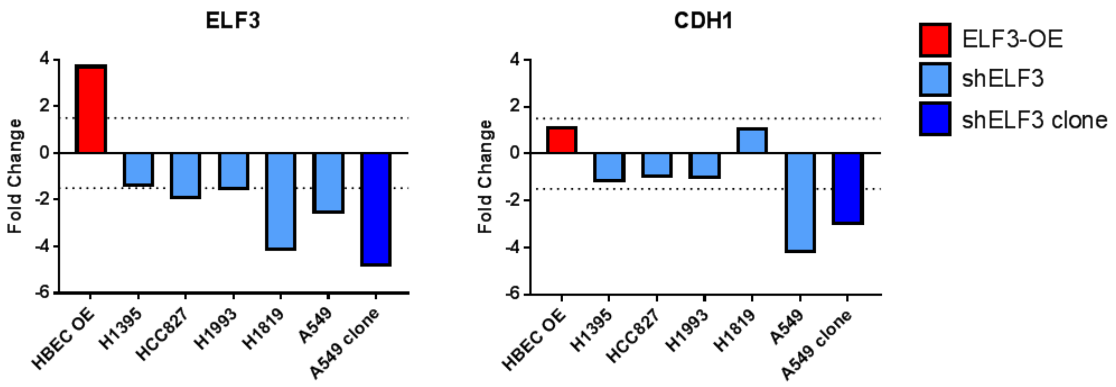

b

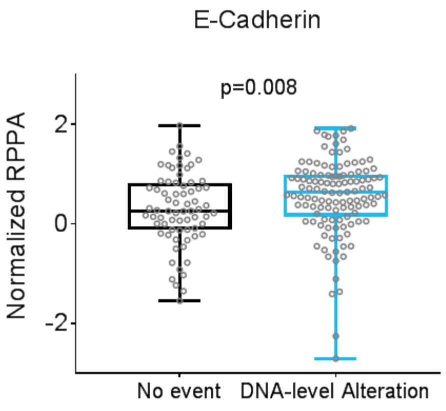

Supplementary Fig. 15. ELF3 expression is positively associated with E-cadherin expression.
(a) Fold change in expression of *ELF3* and *CDH1* in isogenic cell lines as determined by microarray. (b) Box and whiskers plots (center line represents the median, box bounds indicate the 25th and 75th percentiles, and whiskers extend from minimum to maximum) of E-cadherin normalized RPPA expression from the TCGA LUAD dataset that do or do not harbour *ELF3* locus alterations (Mann-Whitney U test).

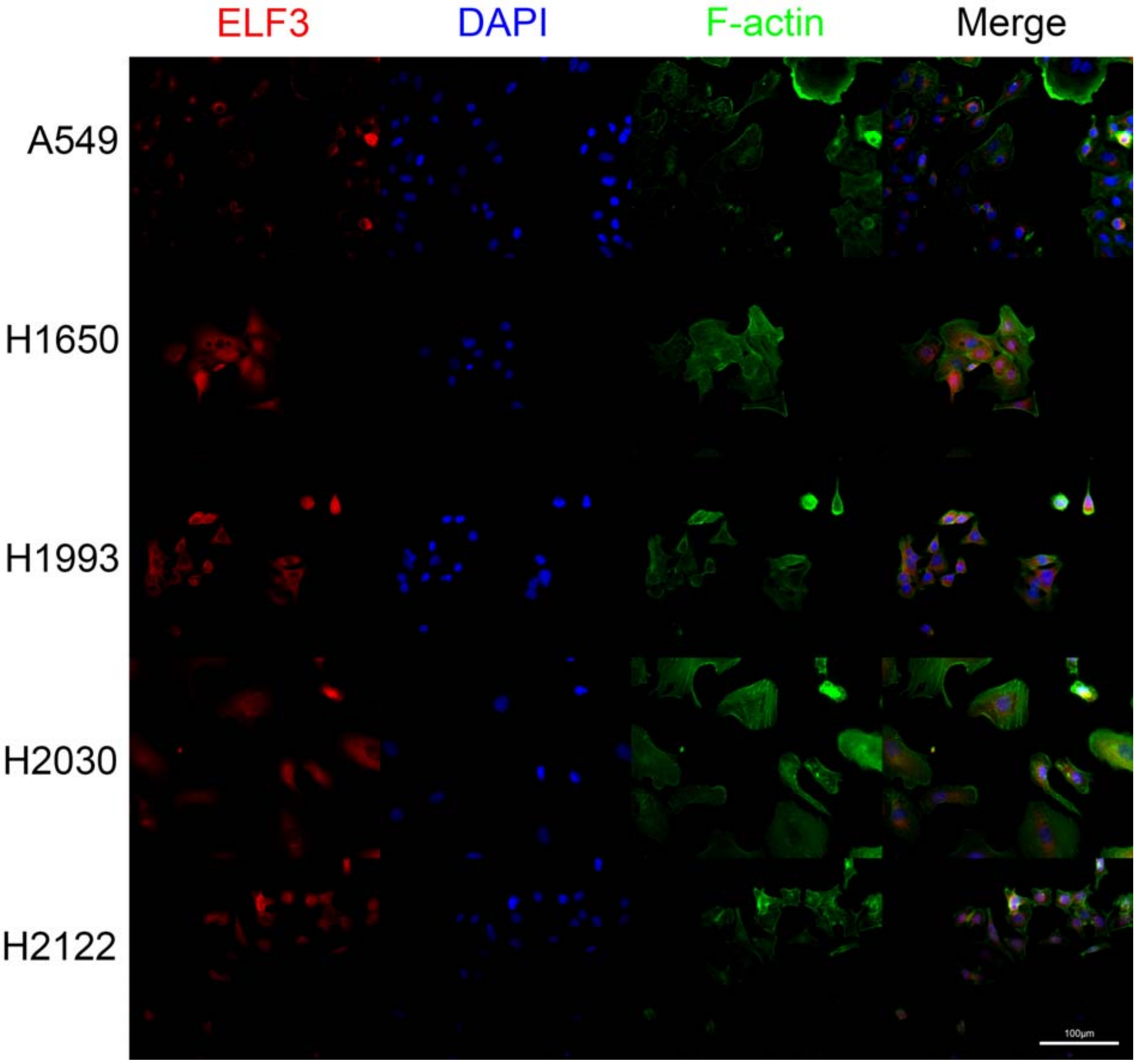

Supplementary Fig. 16. Immunofluorescence imaging of ELF3 subcellular localization in lung adenocarcinoma cell lines.
Representative images of 2D monolayer cultures from five LUAD cell lines (A549, H1650, H1993, H2030, and H2122). Each slide was stained for ELF3 (red) counterstained with DAPI and filamentous actin (blue and green, respectively). Images were captured at 40x magnification (Zeiss Colibri fluorescence microscope, AxioCam MRm camera, AxioVision Rel. 4.8 software) and processed using ImageJ software. Scale bar = 100µm.

Supplementary Table 1. Summary of clinical cohorts.

Cohort	# of samples	TUMOUR		NON-MALIGNANT		Gene Expression Platform
		# LUAD	# LUSC	# LUAD	# LUSC	
BC Cancer Agency (LUAD)*	166	83	0	83	0	Affymetrix GeneChip Human Genome U133 Plus 2.0 Array
BC Cancer Agency (LUSC)	58	0	29	0	29	Illumina HiSeq2000 RNA sequencing
The Cancer Genome Atlas*	1126	513	504	58	51	Illumina HiSeq2000 RNA sequencing
Duke University - GSE3141	111	58	53	0	0	Affymetrix GeneChip Human Genome U133 Plus 2.0 Array
Samsung Medical Center - GSE8894	138	63	75	0	0	Affymetrix GeneChip Human Genome U133 Plus 2.0 Array
Dalhousie University	236	170	66	0	0	Immunohistochemistry
**TOTAL**	**1835**	**887**	**727**	**141**	**80**	

*Multi-omics datasets

Supplementary Table 2. Spearman's correlation of ELF3 expression and lung squamous and adenocarcinoma lineage markers.
RSEM expression of *ELF3* was correlated with the indicated genes across non-small cell lung cancer samples (TCGA, n=1,026).

ELF3 gene pair	Lineage	ρ	95% CI	P-value
KRT8	LUAD	0.317	0.2589 to 0.3727	<0.0001
MUC1	LUAD	0.4667	0.4157 to 0.5148	<0.0001
NKX2-1	LUAD	0.3476	0.2907 to 0.402	<0.0001
TP63	LUSC	-0.3058	-0.3621 to -0.2474	<0.0001
KRT5	LUSC	-0.288	-0.345 to -0.229	<0.0001
SOX2	LUSC	-0.09211	-0.1545 to -0.02902	0.0033
