# Supplementary material for: Epithelial tumor suppressor ELF3 is a lineage-specific amplified oncogene in lung adenocarcinoma
Source: Nat Commun. 2019 Nov 28;10:5438. doi: 10.1038/s41467-019-13295-y (PMC6882813; doi:10.1038/s41467-019-13295-y)
Supplement: Supplementary file 2 — Description of Additional Supplementary Files [file 41467_2019_13295_MOESM2_ESM.pdf]

Description of Additional Supplementary Files

File Name: Supplementary Data 1
Description: **Term enrichment in condition-specific ELF3 protein-protein interaction networks.** The term enrichment tool in pathDIP (version 2.5; http://ophid.utoronto.ca/pathDIP) was used to summarized enriched pathways (corrected q-value < 0.05) across the three pairs of disrupted ELF3 networks (GEO N vs T, TCGA KRASmut ELF3low vs ELF3high, and TCGA KRASwt ELF3low vs ELF3high). A q-value > 0.05 is indicated by -1.

File Name: Supplementary Data 2
Description: **Altered ELF3 interacting partners in lung adenocarcinoma sorted based on the support they have from the three condition-specific networks.**
Sixty-nine ELF3 interacting partners are altered in LUAD in at least one of the three main datasets: GEO, TCGA KRASwt, and TCGA KRASmut. We calculated a combined score for the amount of support they have from these datasets (see Methods). N = normal-specific ELF3 PPI, T = tumour-specific ELF3 PPI, full = full support (significance has passed thresholds), partial = partial support (significance is above or close to threshold, or gene was not found on microarray. If the gene was not present in the gene expression data it is marked with "_". In the total support score, full support = 2, partial support = 1, and no support = 0. In addition, extra support of PPI deregulation in our A549 isogenic model is listed.
