# Supplementary material for: Epithelial tumor suppressor ELF3 is a lineage-specific amplified oncogene in lung adenocarcinoma
Source: Nat Commun. 2019 Nov 28;10:5438. doi: 10.1038/s41467-019-13295-y (PMC6882813; doi:10.1038/s41467-019-13295-y)
Supplement: Supplementary file 3 — Supplementary Data 1 [file 41467_2019_13295_MOESM3_ESM.pdf]

term	frequency (qval<0.05)	GEO_N	GEO_T	KRASwt ELF3low	KRASwt ELF3high	KRASmut ELF3low	KRASmut ELF3high
notch	6	0.0112	0.00181	0.00296	0.00177	0.0111	5.91E-05
signaling	6	1.79E-85	4.51E-78	7.14E-113	7.87E-31	1.70E-81	0.00201
apoptosis	5	0.00193	4.01E-06	1.66E-08	0.00163	3.60E-07	-1
bcr	5	0.0128	0.012	0.00251	0.0494	0.0214	-1
cancer	5	1.55E-11	2.82E-11	1.51E-11	5.92E-07	2.95E-11	-1
cell-cycle	5	2.86E-06	3.37E-05	0.000443	-1	0.0387	0.00295
senescence	5	0.00205	0.000158	0.000288	0.0233	1.21E-05	-1
transcription-factor	5	6.12E-06	7.52E-06	0.000288	0.0233	0.00244	-1
wnt	5	3.14E-06	0.00155	1.03E-05	0.0287	1.66E-06	-1
cascade	4	0.000712	0.000597	5.49E-05	-1	1.95E-05	-1
differentiation	4	3.30E-08	9.78E-06	2.43E-05	-1	0.0296	-1
fas	4	-1	0.012	0.0184	0.0133	0.00288	-1
hypertrophy	4	0.00205	0.000158	0.00311	-1	0.0157	-1
mapk	4	0.000484	6.75E-09	1.07E-11	-1	1.66E-06	-1
myogenesis	4	0.0129	0.000882	0.0179	-1	0.0314	-1
signaling-mediated	4	0.000752	0.000613	0.000109	-1	5.23E-05	-1
smad2-3	4	0.0102	0.00949	0.0138	-1	0.00286	-1
stat	4	0.00474	0.0252	0.0378	-1	0.00552	-1
tcr	4	0.000756	-1	0.00122	0.00209	0.0187	-1
tgf-beta	4	0.00189	9.14E-05	0.000153	-1	7.53E-06	-1
tlr	4	-1	8.76E-05	0.000169	0.0042	3.89E-06	-1
angiogenesis	3	-1	-1	-1	0.0311	0.0408	0.0443
associated	3	-1	0.00501	0.0378	-1	0.00552	-1
bmp	3	0.0102	0.00949	0.0138	-1	-1	-1
constitutive	3	0.0317	-1	0.0432	0.0401	-1	-1
dna-damage	3	0.0273	0.0276	-1	-1	0.0199	-1
estrogen-receptor	3	0.0284	0.0273	-1	-1	0.0113	-1
g1	3	0.00487	0.00468	0.00697	-1	-1	-1
il1	3	-1	0.000524	0.00808	-1	0.000843	-1
integrated	3	-1	-1	-1	0.0311	0.0408	0.0443
jnk	3	-1	0.00149	0.000153	-1	0.000217	-1
nfkb	3	-1	2.52E-06	4.40E-06	-1	0.00552	-1
p38	3	-1	0.0123	0.00311	-1	0.000201	-1
pi3k	3	9.38E-05	0.0116	8.08E-08	-1	-1	-1
polymerase2	3	0.00487	0.031	-1	0.0011	-1	-1
regulation	3	8.73E-06	0.00934	1.85E-11	-1	-1	-1
response	3	0.013	0.00468	-1	-1	0.000881	-1
validated	3	0.00301	0.00301	0.0449	-1	-1	-1
adherens	2	-1	-1	-1	0.0055	-1	0.0039
akt	2	0.00487	-1	0.000423	-1	-1	-1
androgen-receptor	2	0.0284	-1	-1	-1	-1	0.0417
beta-catenin	2	0.0128	0.012	-1	-1	-1	-1
cadherin	2	-1	-1	-1	0.0311	-1	0.0443

cardiac	2	-1	0.00501	0.0378	-1	-1	-1
development	2	-1	0.0278	0.00808	-1	-1	-1
end	2	-1	0.00949	0.0138	-1	-1	-1
excision-repair	2	0.0278	0.0278	-1	-1	-1	-1
gene-expression	2	0.0028	8.23E-05	-1	-1	-1	-1
growth-factor-receptor	2	-1	-1	-1	-1	0.0408	0.0443
jak	2	0.0128	-1	-1	-1	0.0157	-1
joining	2	-1	0.0273	0.0371	-1	-1	-1
junction	2	-1	-1	-1	0.00231	-1	0.000886
notch1	2	-1	-1	0.0151	-1	0.03	-1
organization	2	-1	-1	-1	0.0311	-1	0.0443
promoter	2	0.00189	-1	-1	0.0494	-1	-1
rna	2	1.45E-06	0.00418	-1	-1	-1	-1
signal-transduction	2	0.0202	-1	0.0344	-1	-1	-1
signals	2	-1	0.0312	0.00406	-1	-1	-1
sumoylation	2	0.032	-1	0.0449	-1	-1	-1
targets	2	-1	0.0116	0.0179	-1	-1	-1
tgf-beta-receptor	2	-1	0.0312	0.0449	-1	-1	-1
transcription	2	1.68E-12	8.98E-07	-1	-1	-1	-1
aryl	1	-1	-1	-1	-1	-1	0.0314
atm	1	-1	-1	-1	-1	0.0408	-1
bmp2	1	-1	-1	-1	-1	-1	0.0314
breast	1	-1	-1	-1	-1	-1	0.0443
cell	1	-1	-1	-1	-1	-1	0.0443
cell-adhesion	1	-1	-1	-1	-1	-1	0.0443
controls	1	-1	-1	-1	-1	-1	0.0314
downstream	1	-1	-1	0.0344	-1	-1	-1
ear	1	-1	-1	-1	-1	0.0408	-1
family	1	-1	-1	0.0222	-1	-1	-1
fc-epsilon-receptor1	1	-1	-1	-1	0.0142	-1	-1
gene	1	-1	-1	0.042	-1	-1	-1
glucagon	1	-1	-1	-1	-1	-1	0.0344
hdac	1	-1	-1	-1	-1	0.0408	-1
hiv	1	0.0116	-1	-1	-1	-1	-1
hydrocarbon-receptor	1	-1	-1	-1	-1	-1	0.0314
hypoxia	1	0.0128	-1	-1	-1	-1	-1
igf1r	1	0.0284	-1	-1	-1	-1	-1
il4	1	-1	-1	-1	-1	-1	0.0488
induction	1	-1	-1	0.0181	-1	-1	-1
mast-cells	1	-1	-1	-1	0.0142	-1	-1
molecules	1	-1	-1	-1	-1	-1	0.0488
mtor	1	-1	-1	-1	0.0207	-1	-1
mutant-receptor	1	-1	-1	-1	-1	-1	0.0443
na	1	-1	-1	-1	0.0311	-1	-1
naive	1	-1	-1	-1	0.0142	-1	-1

nascent	1	-1	-1	-1	-1	-1	0.0314
notch2	1	-1	-1	-1	-1	-1	0.0443
p53	1	-1	0.0123	-1	-1	-1	-1
polymerase1	1	0.0129	-1	-1	-1	-1	-1
polymerase3	1	0.0128	-1	-1	-1	-1	-1
protein1	1	-1	-1	-1	-1	-1	0.0314
regulated	1	-1	-1	-1	0.035	-1	-1
role	1	-1	-1	0.0152	-1	-1	-1
stability	1	-1	-1	-1	-1	-1	0.0314
sumo	1	0.0284	-1	-1	-1	-1	-1
t-cells	1	-1	-1	-1	0.0142	-1	-1
tak1	1	-1	-1	0.00134	-1	-1	-1
telomerase	1	-1	0.0273	-1	-1	-1	-1
tlr2	1	-1	-1	-1	-1	0.0408	-1
tlr3	1	-1	-1	-1	-1	0.0408	-1
tlr7	1	-1	-1	0.0371	-1	-1	-1
tlr8	1	-1	-1	0.0371	-1	-1	-1
tlr9	1	-1	-1	0.0138	-1	-1	-1
tnf	1	-1	-1	-1	-1	0.0113	-1
traf6-mediated	1	-1	-1	0.0138	-1	-1	-1
transcriptional-regulation	1	-1	-1	-1	-1	0.0314	-1
transmembrane-receptor	1	-1	-1	-1	-1	-1	0.0314
vegf	1	-1	-1	0.0432	-1	-1	-1
