# Supplementary material for: Epithelial tumor suppressor ELF3 is a lineage-specific amplified oncogene in lung adenocarcinoma
Source: Nat Commun. 2019 Nov 28;10:5438. doi: 10.1038/s41467-019-13295-y (PMC6882813; doi:10.1038/s41467-019-13295-y)
Supplement: Supplementary file 4 — Supplementary Data 2 [file 41467_2019_13295_MOESM4_ESM.pdf]

| Gene Symbol | total support score | GEO | TCGA KRASwt | TCGA KRASmut | extra support for KRASmut (A549) |
|---|---|---|---|---|---|
| ERBB3 | 6 | T_full | Up_full | Up_full | up in ELF3high |
| ETS1 | 6 | N_full | Down_full | Down_full | down in ELF3high |
| TIMP3 | 5 | N_full | Down_part | Down_full | down in ELF3high |
| ARHGEF6 | 4 | | Down_full | Down_full | down in ELF3high |
| CCL11 | 4 | N_full | | Down_full | down in ELF3high |
| CLDN4 | 4 | | Up_full | Up_full | up in ELF3high |
| ELK3 | 4 | | Down_full | Down_full | down in ELF3high |
| ERBB2 | 4 | | Up_full | Up_full | down in ELF3high |
| FLI1 | 4 | | Down_full | Down_full | up in ELF3high |
| GLI2 | 4 | N_full | Down_full | | |
| KDM5B | 4 | T_full | Up_full | | |
| NFKB1 | 4 | N_full | | Down_full | down in ELF3high |
| PMF1 | 4 | | Up_full | Up_full | up in ELF3high |
| ROBO1 | 4 | | Down_full | Down_full | down in ELF3high |
| SLIT2 | 4 | | Down_full | Down_full | down in ELF3high |
| SPDEF | 4 | | Up_full | Up_full | up in ELF3high |
| SPI1 | 4 | N_full | Down_full | | |
| SPINT1 | 4 | | Up_full | Up_full | up in ELF3high |
| TAF9 | 4 | N_full | | Down_full | |
| TRADD | 4 | | Up_full | Up_full | down in ELF3high |
| ZEB1 | 4 | | Down_full | Down_full | down in ELF3high |
| AR | 3 | T_full | | Up_part | |
| INPP5D | 3 | N_full | Down_part | | |
| JUN | 3 | | Up_full | Up_part | |
| TBP | 2 | T_full | _ | _ | _ |
| CDK8 | 2 | T_full | | | |
| CEBPB | 2 | N_full | | | |
| CREBBP | 2 | T_full | | | |
| ELF4 | 2 | | Down_full | | |
| EPHB4 | 2 | | Up_full | | |
| EPS15 | 2 | | | Down_full | down in ELF3high |
| ERF | 2 | | | Up_full | down in ELF3high |
| ETS2 | 2 | | | Up_full | up in ELF3high |
| ETV1 | 2 | | Down_full | | |
| ETV2 | 2 | | | Up_full | down in ELF3high |
| FEM1B | 2 | | Down_full | | |
| FLG | 2 | | Down_full | | |
| FOXO1 | 2 | | Down_full | | |
| FSCN1 | 2 | | Down_full | | |
| GABPA | 2 | | | Down_full | up in ELF3high |
| GANAB | 2 | | | Down_full | down in ELF3high |

| | | | | | |
|---|---|---|---|---|---|
| GIT2 | 2 | | Down_full | | |
| GRB2 | 2 | | Down_full | | |
| H3F3A | 2 | | Up_full | | |
| HOXA5 | 2 | T_full | | | |
| IRF6 | 2 | | Up_full | | |
| JAK1 | 2 | | | Down_full | down in ELF3high |
| KAZALD1 | 2 | | | Up_full | up in ELF3high |
| KRT4 | 2 | | Up_full | | |
| LEF1 | 2 | | Down_full | | |
| MED9 | 2 | | Up_full | | |
| MSN | 2 | | Down_full | | |
| MYC | 2 | N_full | | | |
| NCF1B | 2 | | Down_full | | |
| NCK2 | 2 | T_full | | | |
| NCOA3 | 2 | | | Down_full | down in ELF3high |
| NFKBIZ | 2 | | Up_full | | |
| NKX2-1 | 2 | T_full | | | |
| NLRC3 | 2 | | Down_full | | |
| NOTCH1 | 2 | | Down_full | | |
| PLA2G4A | 2 | | Up_full | | |
| SAMD10 | 2 | | Up_full | | |
| SKI | 2 | N_full | | | |
| SPEN | 2 | N_full | | | |
| SRF | 2 | N_full | | | |
| TARS | 2 | | | Down_full | up in ELF3high |
| TGFB1 | 2 | N_full | | | |
| TGFBR2 | 2 | | Down_full | | |
| UBE2D1 | 2 | | | Down_full | down in ELF3high |
