# Supplementary material for: Epithelial tumor suppressor ELF3 is a lineage-specific amplified oncogene in lung adenocarcinoma
Source: Nat Commun. 2019 Nov 28;10:5438. doi: 10.1038/s41467-019-13295-y (PMC6882813; doi:10.1038/s41467-019-13295-y)
Supplement: Supplementary file 5 — Reporting Summary [file 41467_2019_13295_MOESM5_ESM.pdf]

natureresearch

Corresponding author(s): Katey Enfield

Reporting Summary

Nature Research wishes to improve the reproducibility of the work that we publish. This form provides structure for consistency and transparency in reporting. For further information on Nature Research policies, see Authors & Referees and the Editorial Policy Checklist.

Statistical parameters

When statistical analyses are reported, confirm that the following items are present in the relevant location (e.g. figure legend, table legend, main text, or Methods section).

n/a	Confirmed	
☐	☒	The exact sample size (*n*) for each experimental group/condition, given as a discrete number and unit of measurement
☐	☒	An indication of whether measurements were taken from distinct samples or whether the same sample was measured repeatedly
☐	☒	The statistical test(s) used AND whether they are one- or two-sided<br>*Only common tests should be described solely by name; describe more complex techniques in the Methods section.*
☐	☒	A description of all covariates tested
☐	☒	A description of any assumptions or corrections, such as tests of normality and adjustment for multiple comparisons
☐	☒	A full description of the statistics including central tendency (e.g. means) or other basic estimates (e.g. regression coefficient) AND variation (e.g. standard deviation) or associated estimates of uncertainty (e.g. confidence intervals)
☐	☒	For null hypothesis testing, the test statistic (e.g. *F*, *t*, *r*) with confidence intervals, effect sizes, degrees of freedom and *P* value noted<br>*Give P values as exact values whenever suitable.*
☐	☒	For Bayesian analysis, information on the choice of priors and Markov chain Monte Carlo settings
☐	☒	For hierarchical and complex designs, identification of the appropriate level for tests and full reporting of outcomes
☐	☒	Estimates of effect sizes (e.g. Cohen's *d*, Pearson's *r*), indicating how they were calculated
☐	☒	Clearly defined error bars<br>*State explicitly what error bars represent (e.g. SD, SE, CI)*

Our web collection on statistics for biologists may be useful.

Software and code

Policy information about availability of computer code

Data collection	IID (ver. 4_2017; http://ophid.utoronto.ca/iid) for collection of protein-protein interaction data.
Data analysis	R packages:<br>(1) mbcb (ver. 2.11.0) to perform background correction and probe summarization of microarray expression data.<br>(2) lumi for analysis of methylation data.<br>(3) MAS5 method using Bioconductor package (Affy package version 1.48.0) for normalization of GEO microarray expression data.<br>Partek Genomics Suite 6.5 for SNP6.0 copy number data analysis.<br>GISTIC 2.0 (https://genepattern.broadinstitute.org/) for analysis of focal chromosomal events.<br>BD FACSDiva software for flow cytometry data.<br>pathDIP (version 2.5; http://ophid..utoronto.ca/pathDIP) for pathway analysis<br>IID (ver. 4_2017; http://ophid.utoronto.ca/iid) for analysis of protein-protein interactions<br>Navigator (http://ophid.utoronto.ca/navigator/) for data visualization<br>Kaplan-Meier analysis (http://kmplot.com/analysis/index.php?p=service&cancer=lung)<br>GraphPad Prism Version 6.0

For manuscripts utilizing custom algorithms or software that are central to the research but not yet described in published literature, software must be made available to editors/reviewers upon request. We strongly encourage code deposition in a community repository (e.g. GitHub). See the Nature Research guidelines for submitting code & software for further information.

Data

Policy information about availability of data

All manuscripts must include a data availability statement. This statement should provide the following information, where applicable:

- Accession codes, unique identifiers, or web links for publicly available datasets
- A list of figures that have associated raw data
- A description of any restrictions on data availability

Gene expression data from paired LUAD and NM tissues have been deposited in the GEO repository under the accession code X [hyperlink X]. LUSC and NM RNA-sequencing data has been deposited in the GEO repository under the accession code Y [hyperlink Y]. LUAD copy number SNP6.0 array data have been deposited in the GEO repository under the accession code Z [hyperlink Z]. A549 expression microarray data has been deposited in the GEO repository under the accession code W [hyperlink W].

The gene expression profiles used to construct pan-tissue ELF3 protein-protein interaction networks are available in the GEO repository under the accession codes GSE19383, GSE26910, GSE3744, GSE5764, GSE20437, GSE5462, GSE6883, GSE9574, GSE9750, GSE20916, GSE8671, GSE41258, GSE5364, GSE11024, GSE14762, GSE21816, GSE7023, GSE8271, GSE6280, GSE6344, GSE781, GSE29721, GSE14520, GSE31908, GSE14407, GSE15578, GSE18520, GSE36668, GSE38666, GSE15471, GSE16515, GSE22780, GSE17951, GSE32448, GSE32982, GSE3325, GSE6956, GSE29265, GSE3467, GSE3678, GSE6004, GSE27155, GSE17025, GSE20347, GSE23400, GSE29001, GSE30784, GSE31056, GSE33426, GSE38129, GSE53757, GSE64985, GSE7305, GSE7307, GSE7803; gene expression profiles used to construct ELF3 protein-protein interaction networks in non-small cell lung cancer are available under the accession codes GSE31210, GSE10245, GSE19188, GSE28571, GSE31908, GSE7670, GSE10072, GSE5364 (https://www.ncbi.nlm.nih.gov/geo/).

Other datasets referenced during the study are available from the TCGA Data Portal [https://tcga-data.nci.nih.gov/tcga/tcgaDownload.jsp], deposited in GEO under the accession numbers GSE3141 ([https://www.ncbi.nlm.nih.gov/geo/query/acc.cgi?acc=GSE3141] and GSE8894 [https://www.ncbi.nlm.nih.gov/geo/query/acc.cgi?acc=GSE8894], and the Kaplan-Meier Plotter Lung Cancer webpage (http://kmplot.com/analysis/index.php?p=service&cancer=lung). All the other data supporting the findings of this study are available within the article and its Supplementary Information files and from the corresponding author upon reasonable request. A reporting summary for this article is available as a Supplementary Information file.

Field-specific reporting

Please select the best fit for your research. If you are not sure, read the appropriate sections before making your selection.

☒ Life sciences ☐ Behavioural & social sciences ☐ Ecological, evolutionary & environmental sciences

For a reference copy of the document with all sections, see nature.com/authors/policies/ReportingSummary-flat.pdf

Life sciences study design

All studies must disclose on these points even when the disclosure is negative.

Sample size	For analysis of clinical specimens of lung cancer and non-malignant lung tissue, we accessed numerous large datasets of non-small cell lung cancer. BCCA LUAD n=83 LUSC n=29. TCGA LUAD n=513 LUSC n=504. Duke University LUAD n=58 LUSC n=53. Samsung Medical Centre LUAD n=63 LUSC n=75. Dalhousie University TMA LUAD n=170 LUSC n=66.
Data exclusions	No data were excluded.
Replication	All in vitro experiments are the result of at least three independent experiments. All experimental replicates were in agreement.<br>In vivo experiments were performed once but in 12, 24, and 8 mice, due to cost restrictions.
Randomization	Patients were grouped by ELF3 status (at the DNA, RNA, or protein level). We did not find any clinical variables to be significantly associated with ELF3 status by Multivariate ANOVA (p>0.05).<br>For in vitro and in vivo experiments, isogenic cell lines were used to control for genetic variability between cell lines.
Blinding	The pathologist was blinded to patient and clinical information when scoring ELF3 staining by immunohistochemistry.<br>For all other analysis, patients were intentionally selected based on expression or disruption of ELF3 and therefore blinding was not relevant in this case.

Reporting for specific materials, systems and methods

Materials & experimental systems

n/a	Involved in the study	
☐	☒	Unique biological materials
☐	☒	Antibodies
☐	☒	Eukaryotic cell lines
☒	☐	Palaeontology
☐	☒	Animals and other organisms
☐	☒	Human research participants

Methods

n/a	Involved in the study	
☒	☐	ChIP-seq
☐	☒	Flow cytometry
☒	☐	MRI-based neuroimaging

Unique biological materials

Policy information about availability of materials

Obtaining unique materials	Tumour and non-malignant lung tissues were collected from treatment naive patients at time of surgical resection and frozen in liquid nitrogen. Tissues were obtained from the Tumour Tissue Repository of the British Columbia Cancer Agency or Vancouver General Hospital under informed written patient consent and with approval from the University of British Columbia – BC Cancer Agency (BCCA) Research Ethics Board.

Antibodies

Antibodies used	anti-ELF3 HPA003479 Sigma-Aldrich<br>anti-ESE1 [EPESER1] ab133621 abcam<br>anti-Histone H3 #9715 Cell Signaling Technology<br>phalloidin Invitrogen<br>PerCP-CyTM5.5 Mouse Anti-BrdU antibody, BD Bioscience<br>anti-Annexin V, BD Bioscience
Validation	anti-ELF3 HPA003479 Sigma-Aldrich was compared against several other anti-ELF3 antibodies using xenograft tissue from HCC827 control tumours with qPCR validated expression of ELF3. Staining performance was advised by a pulmonary pathologist.<br><br>anti-ESE1 [EPESER1] ab133621 abcam for western blotting was validated using isogenic cell lines with qPCR validated ELF3 expression.

Eukaryotic cell lines

Policy information about cell lines

Cell line source(s)	The following cell lines have been purchase by ATCC (H1395, HCC827, A549, H1993, H1819), whereas HBEC-KT cells were provided to Dr. Wan Lam by Dr. Adi Gazdar and Dr. John Minna.
Authentication	Provided directly by ATCC.
Mycoplasma contamination	All cell lines tested negative for mycoplasma.
Commonly misidentified lines<br>(See ICLAC register)	n/a

Animals and other organisms

Policy information about studies involving animals; ARRIVE guidelines recommended for reporting animal research

Laboratory animals	6-8 week old male NOD-SCID and NRG mice.
Wild animals	The study did not involve wild animals.
Field-collected samples	The study did not involve samples collected in the field.

Human research participants

Policy information about studies involving human research participants

Population characteristics	BCCA: Median age 69, 57% stage I, 29% stage II, 11% stage III, 3% stage IV, 44% current smokers, 32% former smokers, 24% never smokers<br>TMA: Median age 67, 50% stage I, 28% stage II, 21% stage III, 1% stage IV, 30% current smokers, 59% former smokers, 6% never smokers<br>TCGA LUAD: Median age 66, 54% stage I, 24% stage II, 16% stage III, 6% stage IV, 23% current smokers, 60% former smokers,

	14% never smokers<br>TCGA LUSC: Median age 68, 50% stage I, 31% stage II, 17% stage III, 2% stage IV, 86% current or former smokers
Recruitment	This study involved retrospectively analyzed samples and as such patients were not recruited.

Flow Cytometry

Plots

Confirm that:

☒ The axis labels state the marker and fluorochrome used (e.g. CD4-FITC).

☒ The axis scales are clearly visible. Include numbers along axes only for bottom left plot of group (a 'group' is an analysis of identical markers).

☒ All plots are contour plots with outliers or pseudocolor plots.

☒ A numerical value for number of cells or percentage (with statistics) is provided.

Methodology

Sample preparation	Cell apoptosis was quantified using the BD PharmingenTM Annexin V Apoptosis Detection Kit I according to the manufacturer's instructions (BD Bioscience, Mississauga, ON, Canada). Cells were grown in complete or serum free media for 72 hours prior to cell processing, staining, and analysis by flow cytometry .<br><br>Cell proliferation was quantified using the BD PharmingenTM Apoptosis, DNA Damage and Cell Proliferation Kit (BD Bioscience, Mississauga, ON, Canada). Cells were incubated with BrdU for 8-24h, and processed according to the manufacturer's instructions. Briefly, cells were fixed, permeabilized, and treated with DNase I before staining with PerCP-CyTM5.5 Mouse Anti-BrdU antibody and DAPI (1µg/ml).
Instrument	Cells were analyzed using the BD FACS CantoTM II cell analyzer (BD Bioscience, Mississauga, ON, Canada).
Software	FACSDiva Software
Cell population abundance	Flow cytometry experiments were performed on pure populations of isogenic cell lines.
Gating strategy	Cells were gated on live singlets. For proliferation assays, cells untreated with BrdU but stained with anti-BrdU antibody (BD Bioscience) were used as a gating control.

☒ Tick this box to confirm that a figure exemplifying the gating strategy is provided in the Supplementary Information.
